# Updated Taxonomy of *Iris scariosa* (Iridaceae) Inferred from Morphological and Chloroplast DNA Sequence Data with Remarks on Classification of *Iris* subg. *Iris*

**DOI:** 10.3390/plants13172349

**Published:** 2024-08-23

**Authors:** Eugeny V. Boltenkov, Elena V. Artyukova

**Affiliations:** 1 Botanical Garden-Institute, Far Eastern Branch, Russian Academy of Sciences, 690024 Vladivostok, Russia; 2 Federal Scientific Center of the East Asia Terrestrial Biodiversity, Far Eastern Branch, Russian Academy of Sciences, 690022 Vladivostok, Russia; artyukova@biosoil.ru

**Keywords:** chloroplast DNA, infrageneric classification, subgenus *Iris*, molecular phylogeny, morphological characters, nomenclature, pollen, taxonomy

## Abstract

*Iris scariosa* is a rhizomatous perennial whose taxonomy and distribution range still remain unclear. The results of our examination of literature, specimens, and wild plants have shown that *I. glaucescens*, described from Kazakhstan, and *I. timofejewii*, considered to be endemic to the Republic of Dagestan, Russia, are very closely related to *I. scariosa*. We have carried out molecular phylogenetic analyses for the first time to clarify the taxonomy of *I. scariosa*. For this, we sequenced six chloroplast DNA regions of an extended sampling that comprised the accepted species *I. glaucescens* and *I. timofejewii*, which has revealed their strong affinity to the accession of *I. scariosa* from the vicinity of Astrakhan, Russia. A thorough revision of the morphological characters has confirmed the lack of evident differences between *I. scariosa* and *I. timofejewii*. Thus, the analyses support a broad species circumscription of *I. scariosa*. We here reduce *I. timofejewii*, as well as *I. curvifolia*, considered to be endemic to Xinjiang, western China, to synonymy of *I. scariosa*. Color illustrations, updated nomenclature, and data on distribution of *I. scariosa* are provided. A lectotype for *I. astrachanica* and a neotype for *I. timofejewii* are designated here. Also, the phylogenetic relationships within *I*. subg. *Iris* are outlined, and an updated classification of the subgenus is proposed. We have recovered six major lineages within four major clades which we recognize as sections. Here, we propose two new nomenclatural combinations, a revised taxonomic treatment, and a new identification key to *I*. subg. *Iris*.

## 1. Introduction

When carrying out studies in the framework of the taxonomic research on the genus *Iris* L. (Iridaceae) in Russia, we paid attention to the still unresolved taxonomy and distribution range of *I. scariosa* Willd. ex Link. It is a rhizomatous perennial distributed in Eurasia. The name *I. scariosa* was described by Link without indication of the collection locality [1]. It was considered as originally described from specimens collected in Siberia [2,3] or near the Caspian Sea, from the Volga River estuary [4,5,6,7].

**Figure 1:**
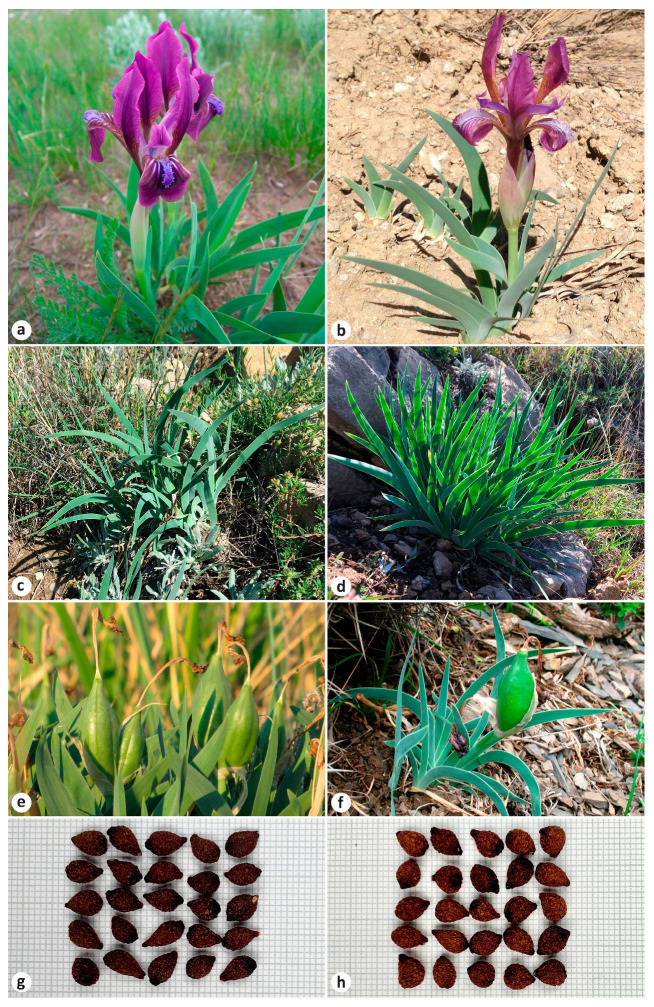
Morphology of the *Iris* species studied: (**a**) *I. scariosa*, habit (Russia, Stavropol Krai, vicinity of Arzgir Village); (**b**) *I. timofejewii*, habit (Russia, Republic of Dagestan, Gunib Village); (**c**,**d**) *I. timofejewii*, leaf morphology within a population (Russia, Republic of Dagestan, vicinity of Botlikh Village); (**e**) *I. scariosa*, in fruiting (Russia, Altai Krai, vicinity of Lake Kuchuk); (**f**) *I. timofejewii*, in fruiting (Russia, Republic of Dagestan, vicinity of Rutul Village); (**g**) *I. scariosa*, seeds (Russia, Altai Krai, vicinity of Lake Kuchuk); (**h**) *I. timofejewii*, seeds (Russia, Republic of Dagestan, vicinity of Tsudakhar Village). Photos by (**a**) S. Banketov, (**b**–**d**,**g**,**h**) E. Boltenkov, (**e**) A. Grebenjuk, and (**f**) *A. Teymurov*.

*Iris scariosa* is distinguished by its membranous bracts and extremely glaucous leaves (Figure 1a). The latter feature was noticed by Bunge when he gave the plant the name *I. glaucescens* Bunge [8]. It was described from plants collected in the East Kazakhstan Region, Kazakhstan [8] (p. 58). Due to their morphological similarity, *I. glaucescens* was considered a synonym of *I. scariosa* for almost a century [2,4,5,9,10,11,12,13,14,15,16].

Rodionenko [15] assumed that *I. scariosa* was a hybrid between *I. pumila* L. and *I. glaucescens*. Shevchenko [6] believed that there were some morphological differences between the two species, however, without specifying them, and resurrected *I. glaucescens* from *I. scariosa*. She also noted that *I. scariosa* had a small distribution range in the Eastern Ciscaucasia and the Lower Volga, while *I. glaucescens* had a wide distribution range, covering the southern part of the West Siberian Plain, the Kazakh Uplands, Altai, the Lake Balkhash area, Dzungaria, and northwestern Mongolia. Subsequent authors believed that *I. glaucescens* was common in Kazakhstan and Western Siberia, and the closely related *I. scariosa* was an endemic to the lower reaches of the Volga and Don rivers [7,17]. Both species are currently accepted with their respective distributions [18,19,20,21,22,23,24,25,26,27,28,29,30].

In addition, it has been suggested that *I. timofejewii* Woronow (Figure 1b) is likely a form of *I. scariosa* [14]. Both species have the same chromosome number, 2*n* = 24 [31,32]. Rodionenko [15] reported *I. timofejewii* as closely related to *I. scariosa* but differing in the type of pollen grain ornamentation. Currently, *I. timofejewii* is considered endemic to the Republic of Dagestan, Russia [18,19,20,21,22,24,25,26,27,30,33] and is listed as an endangered species [34,35].

The major aim of our study was to provide an updated molecular phylogeny of *I. scariosa* based on an extended sampling that included accessions from the recorded distribution ranges of *I*. *glaucescens* and *I. timofejewii*, i.e., from Kazakhstan and the Republic of Dagestan, Russia, respectively. In this study, we also aimed to examine the morphological characters of *I. scariosa* and related species, verify the pollen morphology using scanning electron microscopy, and propose a revised taxonomy of *I. scariosa* with both molecular and morphological evidence taken into account. The taxon sampling for the present molecular study also included representatives of all sections of *I*. subg. *Iris* according to references [16,36,37]. In this regard, an additional aim was to resolve phylogenetic relationships between the sections in *I*. subg. *Iris* on the basis of a plastid sequence dataset for clarifying the classification of the subgenus.

## 2. Materials and Methods

### 2.1. Molecular Study

#### 2.1.1. Taxonomic Sampling

The taxon sampling focused on the species of *I*. sect. *Iris*. We attempted to provide as extensive sampling as possible. One of us (E.V. Boltenkov) made botanical expeditions to Armenia in 2015, Kyrgyzstan in 2022, and the Republic of Dagestan, Rostov Oblast, and Stavropol Krai, Russia, in 2015, 2022, and 2023. Also, we used material collected from Bulgaria, Kazakhstan, Tajikistan, Uzbekistan, and Russia by our colleagues and ensured that all accessions were fully verified. The complete list of the examined taxa, including information on samples, is provided in Table 1.

**Figure 2:**
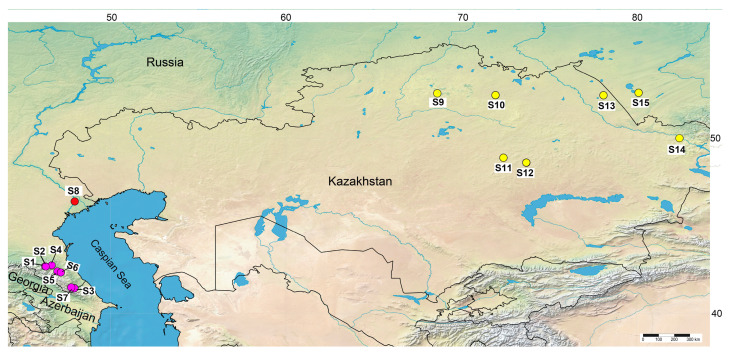
Map showing the geographical origins of the *Iris scariosa* (red dot), *I. timofejewii* (violet dots), and *I. glaucescens* (yellow dots) samples analyzed in the present study (composed using https://www.simplemappr.net, CC 1.0; accessed on 5 April 2024). For locality codes, see Table 1.

The main set of samples for the present study included 15 accessions representing *I. timofejewii* from the mountainous central and southern part of the Republic of Dagestan (S1–S7) where this species is distributed according to the literature and dedicated websites [22,23,33], *I. scariosa* from Russia (S8), and *I. glaucescens* from Kazakhstan (S9–S14) and Altai Krai, Russia (S15). Two accessions (S1 and S2) were obtained from Botlikhsky Raion (formerly the Andiysky Okrug) of the Republic of Dagestan, Russia, the type locality of *I. timofejewii* [43]. The accession S8 originally comes from the Volga River estuary in the vicinity of Astrakhan, Russia, the alleged type locality of *I. scariosa* according to references [6,7]. The accession S14 was collected near the type locality of *I. glaucescens* in the vicinity of Ust-Kamenogorsk (or Oskemen), Kazakhstan. The sampling localities for accessions S1–S15 are shown in Figure 2.

The taxon sampling included the following species from all sections of *I*. subg. *Iris* according to references [16,36]: *I. aphylla* L. (four accessions, A1–A4) and *I. pumila* (two accessions, P1 and P2) and one accession each of *I. alberti* Regel, *I. imbricata* Lindl., and *I. reichenbachii* Heuff. from *I*. sect. *Iris*; *I. acutiloba* C.A.Mey. and *I. iberica* M.Bieb. (two accessions, Ar1 and Ar2) from *I.* sect. *Oncocyclus* (Siemssen) Baker; *I. longiscapa* Ledeb. (two accessions, Uz1 and Uz2) from *I*. sect. *Hexapogon* (Bunge) Baker; and *I. korolkowii* Regel and *I. stolonifera* Maxim. from *I.* sect. *Regelia* Lynch. The study also included accessions of the previously investigated species [39]: *I. bloudowii* Ledeb., *I. humilis* Georgi, *I. potaninii* Maxim., *I. tigridia* Bunge, and *I. vorobievii* N.S.Pavlova from *I.* sect. *Psammiris* (Spach) J.J.Taylor and *I. goniocarpa* Baker and *I. thoroldii* Baker (sub *I. potaninii* var. *ionantha* Y.T.Zhao) from *I*. sect. *Pseudoregelia* Dykes. Outgroups consisted of *I. dichotoma* Pall. and *I. domestica* (L.) Goldblatt et Mabb. from *I*. subg. *Pardanthopsis* (Hance) Baker that formed a sister clade to *I*. subg. *Iris* [37] and 12 species that represented four series of *I*. subg. *Limniris* (Tausch) Spach.

#### 2.1.2. DNA Extraction, Amplification, and Sequencing

Total genomic DNA was isolated as previously described [44] from silica-dried leaf materials collected in the field, obtained from living collections or taken from herbarium specimens deposited at LE and TASH (herbarium codes according to reference [38]).

Six plastid markers were used. Four intergenic spacers (IGS) of chloroplast DNA (cpDNA), *trnH-psbA*, *rps4-trnS*^GGA^, *trnS-trnG*, and *trnL-trnF* were sequenced for samples from all the sections of *I*. subg. *Iris*, except for the species of *I*. sect. *Psammiris* and *I*. sect. *Pseudoregelia*, for which these regions had already been sequenced previously [39]. Amplification and sequencing of these IGS were carried out according to reference [45]. In addition to these markers, partial sequences of the plastid genes *ndhF* (ca. 2150 bp) and *ycf1* (ca. 1030 bp) were amplified and sequenced following the protocols described by Wilson [46,47]. The *ndhF* and *ycf1* genes were shown to be useful in resolving phylogenetic relationships between species of the genus *Iris* [37,46,48].

The cycle sequencing reactions were performed on both strands as described in references [45,46,47], and sequencing products were separated on an ABI 3130 genetic analyzer (Applied Biosystems, Bedford, MA, USA) at the Joint-Use Center “Biotechnology and Genetic Engineering”, Federal Scientific Center of the East Asia Terrestrial Biodiversity, Far Eastern Branch, Russian Academy of Sciences (FSC EATB FEB RAS), Vladivostok, Russia. Forward and reverse sequences for each region were assembled using Staden Package version 1.4 [49]. All newly generated sequences were deposited in the GenBank database (Table 1). The accession numbers for all the sequences used in the study are listed in Table 1. The sequence data of the *ycf1* and/or *ndhF* plastid genes for *I. dichotoma*, *I. imbricata*, and the *I.* sect. *Pseudoregelia* and *I.* ser. *Laevigatae* (Diels) G.H.M.Lawr. species were accessed from GenBank (Table S1). In addition, sequences of six cpDNA regions were retrieved from the complete plastid genomes of the following *I*. subg. *Iris* species available in GenBank (https://www.ncbi.nlm.nih.gov/, accessed on 15 March 2024): *I. germanica* L. and *I. lutescens* Lam. from *I*. sect. *Iris*, *I. sprengeri* Siehe, *I. lycotis* Woronow, *I*. *haynei* Baker, and *I. gatesii* Foster from *I.* sect. *Oncocyclus*, *I. afghanica* Wendelbo and *I*. *hoogiana* Dykes from *I*. sect *Regelia*, and *I. sichuanensis* Y.T.Zhao (is a synonym of *I. leptophylla* Lingelsh.) from *I*. sect. *Pseudoregelia* (Table S1).

#### 2.1.3. Sequence Alignment and Phylogenetic Analysis

The sequences of each cpDNA region obtained in this study, together with those retrieved from the chloroplast genomes of nine species belonging to *I*. sect. *Iris*, *I*. sect. *Oncocyclus*, *I*. sect. *Regelia*, and *I*. sect. *Pseudoregelia* (Table S1), were aligned manually in SeaView version 4 [50] using the CLUSTAL algorithm and concatenated for each accession. We included indels and length variation in mononucleotide repeats in the dataset because the repeatability tests allowed for removing of PCR errors. Based on this dataset, which also included the *I. dichotoma* sequences as outgroups, haplotypes were identified using DnaSP version 5 [51]. This program was also used to calculate the degree of cpDNA sequence divergence within and between sections of *I*. subg. *Iris* based on nucleotide substitutions.

The haplotype network was constructed by the median-joining method (MJ) with default settings as applied in Network version 4.6 [52]. An 8 bp inversion within the *trnH-psbA* spacer and each deletion/insertion, regardless of size, were treated as a single mutational event.

Phylogenetic relationships among irises were assessed by the maximum parsimony (MP) and maximum likelihood (ML) methods as implemented in PAUP version 4.0 b10 [53] and also by the Bayesian inference method (BI) in MrBayes version 3 [54] via the CIPRES portal [55]. The dataset for these analyses included the sequences of 47 accessions from six recognized sections of *I*. subg. *Iris* and the sequences of 12 species from four series of *I*. subg. *Limniris* (*I*. ser. *Lacteae* Doronkin, *I*. ser. *Laevigatae*, *I*. ser. *Sibiricae* (Diels) G.H.M.Lawr., and *I*. ser. *Ruthenicae* (Diels) G.H.M.Lawr.), and two species from *I*. subg. *Pardanthopsis* (*I. dichotoma* and *I. domestica*) as outgroups. For the MP method, optimal trees were found using a heuristic search with 1000 random addition sequence replicates, starting trees obtained via stepwise addition, TBR branch swapping, and the MUL-Trees option in effect. For the ML and BI methods, the GTR + I + G model was selected according to the Akaike information criterion using MODELTEST version 3.6 [56]. ML heuristic searches were performed with the resulting model settings, 100 replicates of random sequence addition, TBR branch swapping, and the MUL-Trees option. For the BI method, using the default prior settings, two parallel MCMC runs were carried out for 10 million generations, with sampling every 1000 generations for a total of 10,000 samples. The convergence of the two chains was assessed, and posterior probabilities (PP) were calculated from the trees sampled during the stationary phase. The robustness of nodes in the ML and MP trees was tested using bootstrap with 1000 replicates (bootstrap percentage, BP).

### 2.2. Morphological Study

#### 2.2.1. Plant Morphology

In order to clarify the differences between *I. scariosa* and *I. timofejewii*, we selected 31 qualitative and quantitative macromorphological characters: (1) rhizome shape; (2) rhizome diameter; (3) rosette leaf shape; (4) rosette leaf texture; (5) rosette leaf apex shape; (6) rosette leaf surface; (7) rosette leaf length (measured from the base to the apex of the longest rosette leaf); (8) rosette leaf width (when dry, measured at the broadest part of the broadest rosette leaf); (9) stem height (measured from the base of the flowering stem to the base of the outer bract); (10) number of cauline leaves; (11) cauline leaf length (measured from the base to the apex of the upper cauline leaf); (12) number of bracts; (13) number of bracteoles; (14) bract shape; (15) bract texture; (16) bract length (measured from the base to the apex of the outer bract); (17) pedicel length; (18) perianth tube length (measured from the ovary apex to the base of the outer perianth segment, i.e., fall); (19) number of flowers; (20) flower color; (21) flower diameter; (22) fall shape; (23) standard (i.e., inner perianth segment) shape; (24) fruit shape; (25) fruit texture; (26) fruit length; (27) fruit diameter; (28) seed shape; (29) seed color; (30) seed length; and (31) seed diameter.

We obtained the scores of the characters for *I. scariosa* and *I. timofejewii* from our own observations of herbarium specimens at ALTB, LE, MHA, MW, NENU, NS, NSK, PALE, RWBG, TK, UBA, UBU, and VBGI, including the original material for the names studied, and through the database of specimens [57] (see Table S2 for more details). We measured rhizome diameter, fruit length and diameter, and seed length and diameter in the dry state with a digital Vernier caliper Series 532 (Mitutoyo, Aurora, IL, USA). We also examined living plants of *I. timofejewii* in the wild, including at the type locality. The terminology used in the morphological description follows reference [58].

#### 2.2.2. Pollen Morphology

For describing the pollen morphology, we used mature anthers from the herbarium specimens collected in Russia: (1) Astrakhan Oblast, Astrakhan, s.d., *S. Korzhinski s.n*. (LE01015406); (2) Republic of Dagestan, Gunib Village, 27 April 2015, *Boltenkov s.n*. (VBGI); (3) Orenburg Oblast, Akbulaksky Raion, 4 km north of Pokrovka Village, 11 May 1998, *M.S. Knyazev s.n*. (LE). Anthers and pollen grains were mounted on aluminum stubs and sputter coated with gold in a vacuum chamber Q150T ES (Quorum Technologies Ltd., Lewes, UK). The morphological features of dry pollen grains were studied by scanning electron microscopy (SEM). The SEM micrographs were taken with a high-resolution field emission scanning electron microscope Merlin^TM^ (Carl Zeiss, Oberkochen, Germany) at the Joint-Use Center “Biotechnology and Genetic Engineering”, FSC EATB FEB RAS. The accelerating voltage was set at 5 kV; the emission current was set at 80 pA. We focused primarily on the exine ornamentation of pollen grains. The height of the raised part of the sculpture elements was measured. The pollen terminology is based on reference [59].

### 2.3. Taxonomy and Distribution

We used the conservative taxonomy of *Iris* [2,3,4,5,10,11,16,37,46,60,61,62,63,64]. We treated *I. aphylla* (including a taxonomic synonym, *I. furcata* M.Bieb.) in a broad sense (e.g., [65]). For the nomenclature, we consulted relevant articles and recommendations of the *Shenzhen Code* (hereafter ICN, [66]). In the Taxonomic Treatment section (see below), we compiled information on the distribution of *I. scariosa* from our own field data, the herbarium specimens, relevant literature, and the critically assessed wild localities of *I. glaucescens*, *I. scariosa*, and *I. timofejewii* from dedicated websites [22,23,27,57,67]. Since there are no available images of *I. curvifolia* Y.T.Zhao in botanical databases [22,57,67], the taxonomy of the name was based on a comprehensive study of the protologue [68], including the original material deposited at NENU (NENU00014010!–NENU00014012!), a single specimen represented in the Chinese botany databases (XJA00065947; see http://www.nsii.org.cn/2017/specimen.php?id=15469163, accessed on 24 July 2024), and the relevant descriptions and illustrations available from references [3,62,68,69,70,71]. For the typification, we examined the herbarium specimens deposited at BAK, BM, E, ERE, LE, NENU, TBI, and TGM personally or via the databases (B, HAL, L, and P). The accepted names and nomenclatural novelties are highlighted in bold.

## 3. Results

### 3.1. Genetic Divergence and Phylogenetic Relationships within Iris subg. Iris

In the study, we used six cpDNA region sequences from 38 accessions of 20 *I*. subg. *Iris* species: 7 accessions of *I. glaucescens*; 7 accessions of *I. timofejewii*; 3 accessions of *I. aphylla*; 2 accessions of each of *I. iberica*, *I. longiscapa*, and *I. pumila*; and 1 accession of each of *I. acutiloba*, *I. alberti*, *I. bloudowii*, *I. goniocarpa*, *I. humilis*, *I. imbricata*, *I. korolkowii*, *I. potaninii*, *I. reichenbachii*, *I. scariosa*, *I. stolonifera*, *I. thoroldii*, *I. tigridia*, and *I. vorobievii*. Our major goal was elucidation of relationships among *I. glaucescens*, *I. scariosa*, and *I. timofejewii* to estimate the degree of genetic similarity among these three species and their taxonomic status.

We identified a total of 11 haplotypes among the 15 accessions of *I. glaucescens*, *I. scariosa*, and *I. timofejewii* based on polymorphic sites found across 6938 aligned positions of a combined dataset of six cpDNA regions. Of these, five haplotypes were each found in *I. timofejewii* (H1–H5) and *I. glaucescens* (H7–H11). Three haplotypes (H5, H8, and H10) were found at several localities, sometimes geographically very distant from each other, while the others were unique, i.e., found at a single locality. The accessions of *I. glaucescens*, *I. scariosa*, and *I. timofejewii* did not share any haplotypes. The sequence divergence (*D_XY_*) of cpDNA between plants of these three species was low (Table 2), varying from 0.00052 (between *I. scariosa* and *I. timofejewii*) to 0.00099 (between *I. timofejewii* and *I. glaucescens*).

**Figure 3:**
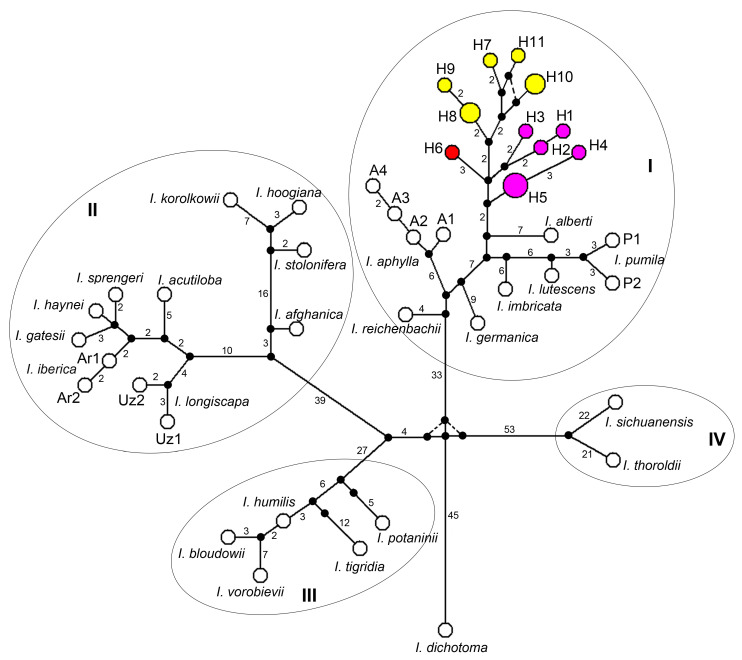
Median-joining network based on cpDNA haplotypes of the *Iris* subg. *Iris* species and *I. dichotoma* as outgroup. Each circle indicates a haplotype, with the size of the circle proportional to the number of localities where this haplotype was found. The 11 haplotypes derived from 15 accessions of *I. scariosa*, *I. glaucescens*, and *I*. *timofejewii* are indicated by colored circles: red, *I. scariosa*; yellow, *I. glaucescens*; violet, *I*. *timofejewii*. Each line between two haplotypes indicates a mutational step, and dashed lines indicate alternative connections of haplotypes. Numerals near the lines connecting haplotypes indicate the number of mutational steps interconnecting two haplotypes (no numeral = one mutation). Small black circles indicate median vectors, inferred by Network version 4.6. Elliptic lines outline the haplotypes representing haploclades I–IV within *I*. subg. *Iris*. For haplotype codes, see Table 1.

The relationships between the haplotypes identified in *I. glaucescens*, *I. scariosa*, and *I. timofejewii* and other representatives of *I*. subg. *Iris*, including those retrieved from the complete plastid genomes of nine species, are shown in Figure 3.

The haplotypes of all the *I*. subg. *Iris* species were interconnected through multiple mutational steps, forming a single network (Figure 3), which was clearly divided into four haploclades. The latter were separated from each other and from *I. dichotoma* haplotype by 65 and more mutational steps. The maximum number of base pair changes was between each haploclade and *I. dichotoma* haplotype (more than 80).

Haploclade I contained all species of *I*. sect. *Iris*. In this haploclade, the *I. glaucescens*, *I. scariosa*, and *I. timofejewii* haplotypes (H1–H11) constituted a separate group that descended from an unsampled or extinct haplotype. In this group, haplotypes were separated from the neighboring ones by several (2–5) mutational steps. The haplotype of *I. alberti* was the closest to this group and connected with the same unsampled haplotype via seven mutational steps. Between two and five mutational steps also separated haplotypes A1–A4 of *I. aphylla*, as well as haplotypes P1 and P2 of *I. pumila*. Multiple mutational steps (more than nine) separated the haplotypes of the studied *I*. sect. *Iris* species from each other, except four steps between the haplotypes of *I. lutescens* and *I. pumila*. The cpDNA sequence divergence between *I. lutescens* and *I. pumila* was estimated at 0.00079, whereas *D_XY_* between other pairs of *I*. sect. *Iris* species ranged from 0.00159 to 0.00389 (Table 2). These values corresponded to *D_XY_* between each species of *I*. sect. *Iris* and the group that included *I. glaucescens*, *I. scariosa*, and *I. timofejewii* (0.00161–0.00320).

Haploclade II contained haplotypes of three sections, *I*. sect. *Hexapogon*, *I*. sect. *Oncocyclus*, and *I*. sect. *Regelia*, while haploclades III and IV contained haplotypes of the species of *I*. sect. *Psammiris* and *I*. sect. *Pseudoregelia*, respectively. The sequence divergence between *I*. sect. *Hexapogon*, *I*. sect. *Oncocyclus*, and *I*. sect. *Regelia* ranged from 0.00181 (between *I*. sect. *Hexapogon* and *I*. sect. *Oncocyclus*) to 0.00465 (between *I*. sect. *Hexapogon* and *I*. sect. *Regelia*), which was considerably lower compared to the divergence between other sections within *I*. subg. *Iris* (Table 3).

**Figure 4:**
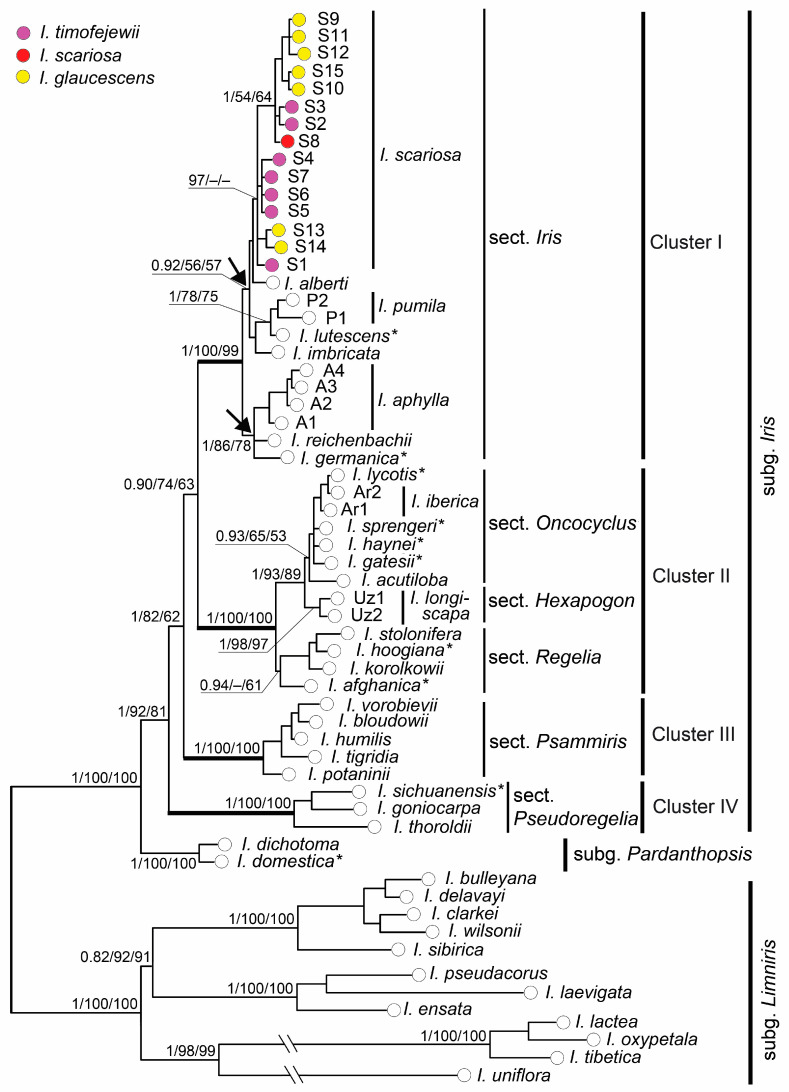
Bayesian majority rule consensus tree for species of the genus *Iris* inferred from combined *trnH*–*psbA*, *rps4–trnS*^GGA^, *trnS*–*trnG*, *trnL*–*trnF*, *ndhF*, and *ycf1* chloroplast data. Asterisks (*) indicate species for which sequences of six cpDNA regions were accessed from GenBank (see Table S1). Numerals above branches are Bayesian posterior probabilities (PP > 0.9) and bootstrap values for the ML and MP methods (BP > 50%). Bold lines indicate branches to four sections of *I*. subg. *Iris,* and arrows indicate two subclades resolved in *I*. sect. *Iris*. The haplotype codes correspond to those listed in Table 1.

The phylogenetic reconstruction methods (BI, ML, and MP) all resulted in basically similar topologies with few differences in statistical support (Figure 4) that were generally consistent with the network topology (Figure 3). As expected, the accessions of all species formed independent clades according to their subgeneric affiliations. *Iris dichotoma* and *I. domestica* formed a sister clade (PP 1.0, BP 100 and 100%) to the monophyletic clade comprising all the *I*. subg. *Iris* species (PP 1.0, BP 92 and 81%).

In the *I*. subg. *Iris* clade, four highly supported clusters were resolved (Figure 4) that corresponded to haploclades revealed by the MJ method (Figure 3). Clusters I, III, and IV included species of *I*. sect. *Iris*, *I*. sect. *Psammiris* (PP 1.0, BP 100 and 100%), and *I*. sect. *Pseudoregelia* (PP 1.0, BP 100 and 100%), respectively. Cluster I (PP 1.0, BP 100 and 99%) included all the studied *I*. sect. *Iris* species which split into two sister groups (Figure 4, see arrows). One of them with moderate support in the ML and MP methods (PP 1.0, BP 86 and 78%) included *I. aphylla*, *I. germanica*, and *I. reichenbachii*. The accessions of *I. glaucescens*, *I. scariosa*, and *I. timofejewii* nested in a second group which also contained the accessions of *I. alberti*, *I. imbricata*, *I. lutescens*, and *I. pumila* and was robust in the BI analysis and weakly supported in the MP and ML methods (PP 0.92, BP 56 and 57%). The latter two species formed a branch with moderate support (PP 1.0, BP 78 and 75%). Another one branch in this group, weakly supported in the ML and MP methods (PP 1.0, BP 54 and 64%), included several accessions of *I. glaucescens* (S9–S11 and S15) and *I. timofejewii* (S2 and S3) together with a single *I. scariosa* (S8) accession (Figure 4).

Cluster II with high support (PP 1.0, BP 100 and 100%) corresponding to haploclade II included all the studied representatives of the three sections of *I*. subg. *Iris*: *I*. sect. *Hexapogon*, *I*. sect. *Oncocyclus*, and *I*. sect. *Regelia*. In this cluster, two sister groups were distinguished: the first group, supported only in the BI and MP methods (PP 0.94 and BP 61%), included species of *I*. sect. *Regelia*; the second group combined species of *I*. sect. *Oncocyclus* and *I*. sect. *Hexapogon* (PP 1.0, BP 93 and 89%). In this group, species of *I*. sect. *Oncocyclus* formed a monophyletic subgroup (PP 0.93, BP 65 and 59%) which was sister to *I. longiscapa* of *I*. sect. *Hexapogon*.

### 3.2. Morphological Study

#### 3.2.1. Macromorphological Comparison

A morphological comparison of *I. scariosa*, including plants from the currently accepted distribution range of *I. glaucescens*, with *I. timofejewii* is listed in Table 4 (also see Table S2 and Figure 1). Both species are variable in rhizome diameter, rosette leaf length and width, flowering stem height, length of the cauline leaf, bract, pedicel, and perianth tube, flower diameter, fruit length and diameter, and also in seed length and diameter. In desert steppes in the Samur River valley (southern Republic of Dagestan) and in Kazakhstan, plants are found with a very dwarf habit (e.g., MW0816525 and MW0816526; see https://plant.depo.msu.ru/module/itemsearchpublic#, accessed on 24 July 2024).

**Figure 5:**
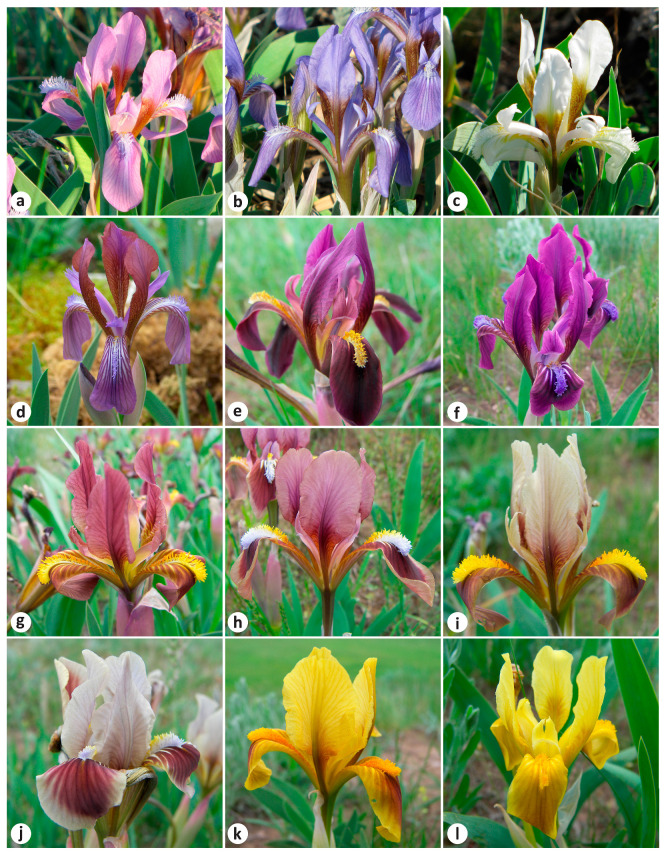
Flower color variation in *Iris scariosa*: (**a**–**c**) Russia, Altai Krai, vicinity of Lake Kuchuk; (**d**) Kazakhstan, Almaty Region, Raiymbek District, Kuluktau Spur; (**e**–**l**) Russia, Stavropol Krai, vicinity of Arzgir Village. Photos by (**a**–**c**) A. Grebenjuk and (**d**–**l**) S. Banketov.

**Figure 6:**
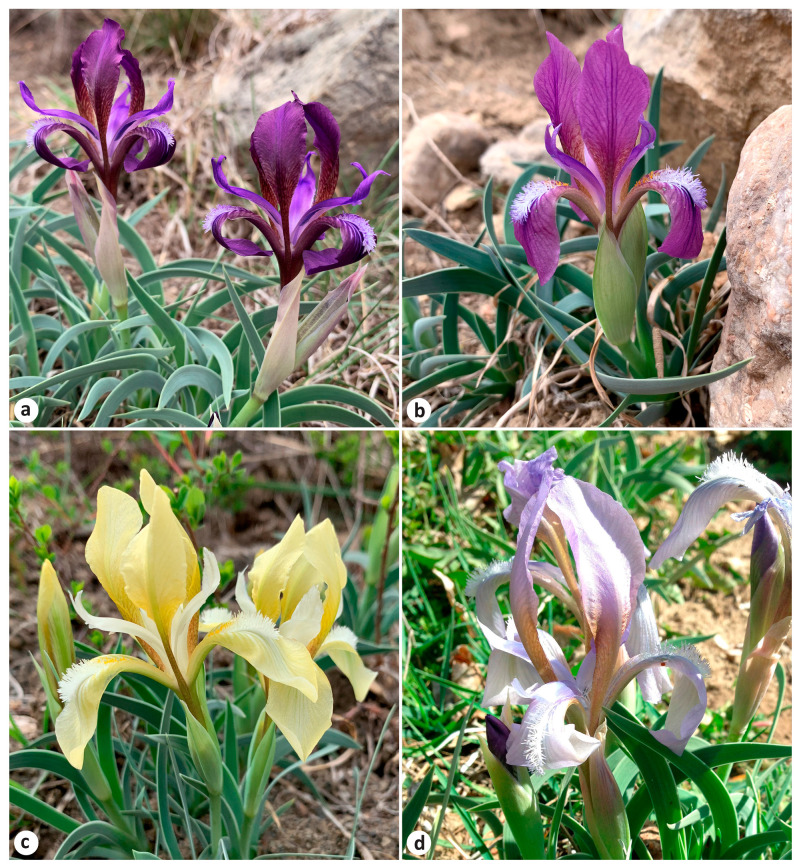
Flower color variation in *Iris timofejewii* (**a**–**d**) within the same locality in Akhtynsky Raion, Republic of Dagestan, Russia (41°25′40.0″ N 47°48′34.0″ E). Photos by E. Boltenkov.

*Iris scariosa* and *I. timofejewii* shared all the qualitative morphological characters (Figure 1). Both had a rhizome that was thick, tough, 0.6–3 cm in diameter, shortly creeping, brownish yellow, covered with short brownish fibers at top; adventitious roots were thickened, yellow-white, with upper and lower parts equal in thickness, and were up to 15 cm long or more. Rosette leaves were ensiform, usually falcate (Figure 1c), or straight under shaded conditions (Figure 1d), chartaceous, with an acute apex and a slightly wider middle, and with leaf sheaths enlarged at rosette base. Rosettes were surrounded by few old leaves, preserved in the form of thin fibers. The flowering stem bore a cauline leaf, 2–5 falcate basal leaves, two bracts, and one bracteole; the inflorescence was two-flowered. Bracts membranous (which refers to the specific epithet *scariosa*) were commonly lilac, broadly lanceolate, with a shortly acute apex. A cauline leaf was located slightly below the middle of the elongated flowering stem (e.g., LE01263915; see http://rr.herbariumle.ru/01263915, accessed on 24 July 2024) or usually at the base of the flowering stem (e.g., LE01263934; http://rr.herbariumle.ru/01263934, accessed on 24 July 2024). The rosette and flowering stem were covered with waxy coating, which was very glaucous (which refers to the specific epithet *glaucescens*), and, thus, grayish green in color. Flowers were variable and were sometimes extremely variable within the same locality, from reddish purple, purple, blue, and light blue, sometimes to almost white, and less often yellow in color (Figure 5 and Figure 6), and were 3.5–5 cm in diameter, borne on short pedicels or sessile. Blade of falls obovate were ca. 5.5 cm long and 2.2 cm wide, folding downwards, and gradually narrowed into a claw, having beard in the form of a central, longitudinal, linear band of hairs; standards were oblanceolate, as long as falls but slightly narrower than falls, and upright. Even beard hairs can be colored in different shades, from yellow to white and blue. Fruit was an oblong-ellipsoid capsule, light brown in color, and up to 8 cm long (e.g., LE01263920; see http://rr.herbariumle.ru/01263920, accessed on 24 July 2024), with a short beak up to 0.5 cm long; pericarp was glabrous and coriaceous, firm, with six distinct ribs, dehiscent very close to the apex (Figure 1e,f). Seeds were pyriform, reddish brown, ca. 8 mm long and 5.5 mm in diameter, with wrinkled surface and without aril (Figure 1g,h).

#### 3.2.2. Pollen Morphology

**Figure 7:**
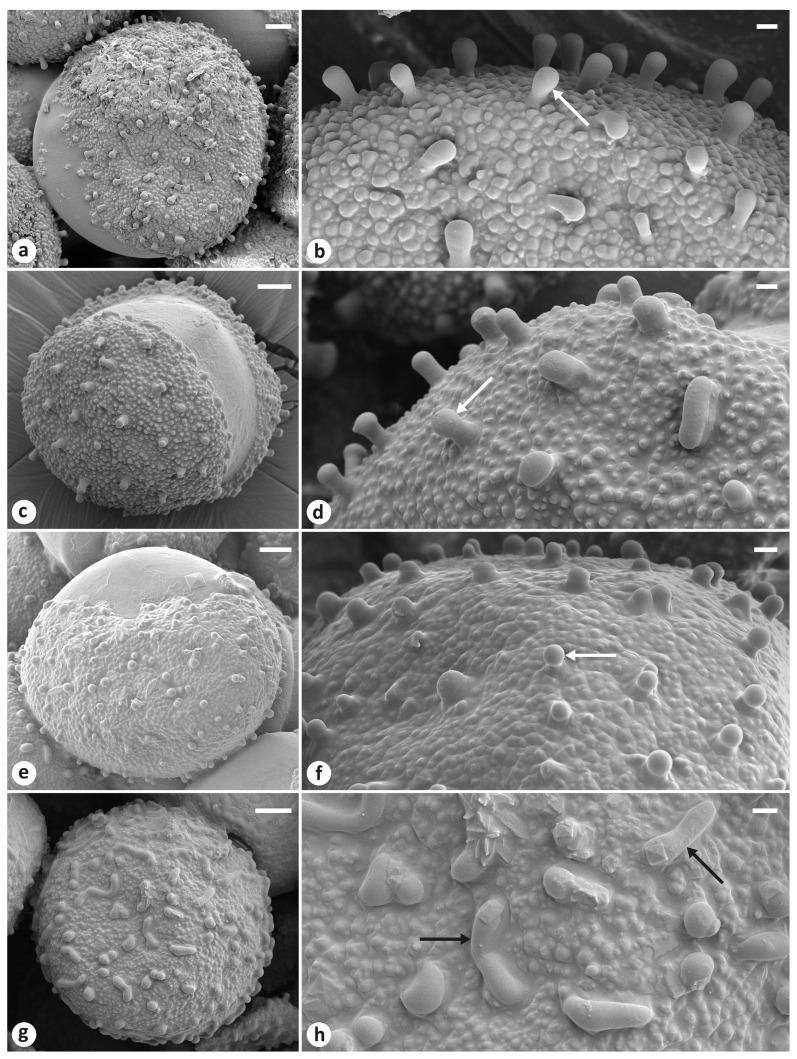
Scanning electron micrographs of dry pollen grains of irises from different localities in Russia: (**a**,**b**) Astrakhan; (**c**,**d**) Republic of Dagestan, Gunib Village; (**e**–**h**) Orenburg Oblast, Pokrovka Village. White arrows indicate papilla; black arrows indicate muri. Scale bars: (**a**,**c**,**e**,**g**) = 10 µm; (**b**,**d**,**f**,**h**) = 2 µm.

The pollen morphological features of the specimens examined in this study by SEM are shown in Figure 7. Below is a general description.

The shape of the pollen grains was oblate spheroidal (Figure 7a,c,e,g). The polar axis ranged from 61 to 91 µm, slightly shorter than the equatorial diameter ranging from 65 to 98 µm. The pollen type was monosulcate (Figure 7a,c,e). The specimen was sulcus distal (anasulcate) and longer than polar axis of pollen grain because it extended over proximal face (Figure 7c), wide (30–70 µm), with sulcus membrane predominantly smooth.

The exine ornamentation of clavate–baculate–granulate type showed that the general surface ornamentation of exine granulate was composed of rounded elements (granula) less than 1 μm in diameter. Free-standing sexine elements were more or less regularly spread over the surface (Figure 7b,d,f) and were club-shaped, with a diameter smaller than the height and thicker at the apex than at the base (clavae), or rod-like, with rounded apices (bacula). The height of these elements in pollen grains of the specimens from Astrakhan, Republic of Dagestan, and Orenburg Oblast was 3.4–4.8, 3.5–4.4, and 1.7–2.5 μm long, respectively. In the specimen from Orenburg Oblast, the surface of the pollen grains located in the anther at its base (Figure 7g,h) contained irregularly arranged, elongated, raised structures (muri) which were not anastomosed, and the pollen grains near the anther apex had a clavate surface (Figure 7e,f).

## 4. Discussion

### 4.1. Taxonomy of Iris scariosa

The protologue of *I. scariosa* is dedicated to the Willdenow Herbarium at Berlin-Dahlem, B [1]. The original material of the name is represented at B by a single specimen (B-W00959010), which is indicated as the lectotype in reference [72]. This specimen is kept in a folder accompanied by a label on which Willdenow handwrote the diagnostic phrase name “*Iris scariosa* …” followed by the synonym “*Iris biflora* Pall.” with a note on geographical origin: “Habitat in Sibiria”. It should be mentioned that, in some cases, Willdenow erroneously indicated the origin on his original labels [73].

Due to the lack of provenance in the protologue of *I. scariosa* [1], several assumptions were made about the origin of the type material (see Section 1). Link reported that Willdenow referred to “*Iris biflora*” from Pallas as *I. scariosa* in the herbarium as follows: “So nennt Willdenow im Herbar. eine *Iris biflora* von Pallas” [1]. According to reference [72] (p. 32), the original material of *I. scariosa* was collected by Pallas as “*Iris biflora*” along the western bank of the Volga River in the vicinity of the fortress of Syzran (now Samara Oblast, Russia) on 8 May 1769. Indeed, Pallas traveled across the Samara region where he collected “*Iris biflora*” near Syzran on 8 May and near the Komarovka Village on 12 May 1769 [74] (p. 171 and 177, respectively). However, *I. scariosa* was (and currently is) not common in the Samara region (see [22,75]). It was confirmed by the specimen that Pallas collected in May 1769 (BM000832596!, “*Iris biflora*, init[io]. Maji floretat, 1769, [*P.S. Pallas*] *s.n.*”; see https://data.nhm.ac.uk/object/9756d97a-8ef4-467e-9c4f-2fd56ca08655, accessed on 24 July 2024). According to Dykes [76] and our results, the specimen BM000832596 is *I. aphylla*, a species currently distributed in Samara Oblast (see [22,75]). Thus, the most likely explanation is that the original material of *I. scariosa* was collected in the vicinity of Astrakhan near the Volga River estuary, as suggested in references [4,5,6,7]. Pallas was in the Astrakhan Governorate, Russia, in late April 1793 [77] (pp. 92–93).

*Iris astrachanica* Rodion. was also described from the vicinity of Astrakhan [15]. Tzvelev [7] regarded *I. astrachanica* as a synonym of *I. scariosa*. Since then, this approach has been accepted [6,18,19,23,25,27,28,29,30,78].

The above data of phylogenetic analyses (Figure 3 and Figure 4) and comparisons of nucleotide divergence levels (Table 2) confirm that *I. scariosa* from Astrakhan; the plants from Altai Krai, Russia, and Kazakhstan, here referred to as *I. glaucescens*; and the plants from the Republic of Dagestan, Russia, here referred to as *I. timofejewii*, are best recognized as a single species for which *I. scariosa* has priority.

In the present study, we did not find diagnostic features to distinguish *I. timofejewii* from *I. scariosa*. Both species demonstrated similar patterns of morphological variability and shared all the qualitative characters (Table 4). The comparative study of plants from the mountainous part of the Republic of Dagestan and *I. scariosa* showed that the foliage and the organs of the flowering stem were very variable, with their morphology depending, to a very large extent, on habitat conditions. The field observations in the Republic of Dagestan clearly showed that in the Samur River valley, on clayey dry soils, plants are dwarf, while in the central, mountainous Dagestan, on loose soils, plants have a habit typical of *I. scariosa*. We found that the shape of rosette leaves depends on light intensity within the same locality: the rosette leaves were falcate in sunlit areas (Figure 1c) and straight under shaded conditions (Figure 1d). It was also noted that, under cultivation, leaves of *I. timofejewii* are usually straight rather than sickle-shaped [15] (p. 257).

Rodionenko [15] noted that *I. scariosa* differs from *I. timofejewii* and *I. astachanica* by the reticulate ornamentation of pollen grains, while in the latter two species, the exine ornamentation is papillate. Nonetheless, the results of the present study clearly show the clavate–baculate–granulate type of exine ornamentation in all pollen samples (Figure 7). Hence, from a morphological viewpoint, all the plants may, in fact, be regarded as a single variable species. We have confirmed that *I. glaucescens* is a synonym of *I. scariosa* and consider *I. timofejewii* to be a synonym of the latter. The same exine pattern is characteristic of *I. adriatica* Trinajstić ex Mitić, *I. attica* Boiss. & Heldr., and *I. pumila* [79,80,81]. These species belong to the group of dwarf bearded irises treated as *I*. ser. *Pumilae* G.H.M.Lawr. [60].

*Iris scariosa* was occasionally in cultivation under the name *I. eulefeldi* Regel [11]. The original material of *I. eulefeldi* was collected along the Talki River in Chinese Dzungaria (see below). It was regarded as a robust variety of *I. scariosa* [10]. Grubov [5] noted that *I. eulefeldi* and typical plants of *I. scariosa* were found growing together in the Tien Shan Mountains (Yining County-level city, or Ghulja) and the Dzungarian Alatau mountain range. *Iris eulefeldi* has long been recognized as a taxonomic synonym of *I. scariosa* [4,5,11,12,14,15,16,19,24,27,29,30]. To the best of our knowledge, these two taxa are identical.

Furthermore, *I. curvifolia* has the same distribution range as *I. eulefeldi*, i.e., north of Yining, northern Xinjiang Uyghur Autonomous Region (Dzungaria), western China. *Iris curvifolia*, considered endemic to China [82], was described from Bole, a county-level city (northern Xinjiang, China) [68], and is distributed only in Xinjiang [3,62,69,83,84]. However, *I. scariosa* is also common in Xinjiang, including Bortala Mongolian Autonomous Prefecture (see [22]), from where *I. curvifolia* was described.

According to the diagnosis, *I. curvifolia* differs from *I. scariosa* by having yellow flowers and ovoid, shortly beaked fruit [68]. However, in the diagnosis of *I. curvifolia*, the features of the flower and fruit completely match those of *I. scariosa* (Figure 1 and Figure 5, Table 4). A comparison of the available herbarium specimens of *I. curvifolia*, including illustrations [68,69,70,71] and the relevant species descriptions available in the literature [3,62,68], to the specimens of *I. scariosa* from China (e.g., HNWP No. 19365, KUN0360536, NAS00555345, PE01013381, and PE01013382; see https://www.cvh.ac.cn/index.php, accessed on 24 July 2024) and from other parts of its distribution range (Table 4) has shown that their features of the rhizome, roots, rosette leaves, flowering stem, bracts, flowers, fruit, and seeds are identical.

The original material of *I. curvifolia* (NENU00014010–NENU00014012) is represented by three plants. Rhizome of *I. curvifolia* is thick, tough, 0.7–1.5 cm in diameter, shortly creeping, brownish yellow, covered at top with short brownish fibers; adventitious roots are thickened, yellow-white, with their upper and lower parts similar in thickness. Rosette leaves are ensiform, falcate or straight, with acute apex and slightly wider middle and with leaf sheaths at the rosette base enlarged, up to 20 cm long and 0.9–1.3 cm wide. Rosette is surrounded by few old leaves. The flowering stem bears the cauline leaf and falcate basal leaves, 8–18 cm tall; two bracts and one bracteole, membranous, bracts broadly lanceolate, apex shortly acute, and outer bract 4–5 cm long; inflorescence two-flowered; perianth tube 2–2.2 cm long. According to references [3,62,68], leaves of *I. curvifolia* are glaucous green; flowers are 4.5–6 cm in diameter; blade of falls are obovate, ca. 4.5 cm long and 1.5 cm wide; standards are oblanceolate, ca. 4 cm long and 1.3 cm wide; capsules are oblong-ellipsoid, with six distinct ribs, ca. 4 cm long and 2 cm wide, shortly beaked at the apex; and seeds are pyriform, reddish brown, ca. 0.7 cm long. Since there are no consistent morphological differences between these species, we suggest that *I. curvifolia* should be considered a synonym of *I. scariosa*.

#### 4.1.1. Taxonomic Treatment of *Iris scariosa*

There are some comments concerning the type citation of the names under study. Below are the details that should be clarified:

(i) On the herbarium sheet at B (B-W00959010; see https://herbarium.bgbm.org/object/BW00959010, accessed on 24 July 2024), which is the current lectotype of *I. scariosa* [72], there are two notes handwritten by Von Schlechtendal: “*Ir. scariosa* 1” and “[collection history]: Pallas. [to] W.[illdenow Herbarium]”. According to reference [73] (p. 344), “if a folder contains more than one sheet, the individual sheets are also sequentially numbered”. Thus, the number “1” means that the folder of *I. scariosa* contained more than one sheet, as in the cases of *I. caricifolia* Pall. ex Link [85] and *I. setosa* Pall. ex Link [86]. It has been established that the label of HAL0109666 was erroneously replaced by that of the specimen HAL0109667 [85] (p. 288), which is the original material of the name *I. scariosa* (see below). The specimen HAL0109666, erroneously reported as an isolectotype of *I. scariosa* [72], is actually an isolectotype of *I. oxypetala* Bunge [85].

(ii) *Iris timofejewii* was described from plants cultivated at the Tiflis Botanical Garden, Tbilisi, Georgia [43]. These plants were raised from the rhizomes collected by Alexander Alfonsovich Grossheim in Andiyskiy Okrug (now Botlikhsky Raion, western Republic of Dagestan, Russia) in 1915. Fedtschenko [2] (p. 549) noted that no original material for *I. timofejewii* was known. Our attempts to find the original material for this name in the framework of the present study have not been successful as well. Consequently, neotypification is required according to the Art. 19.11 of the ICN. For this purpose, LE01268154 is here designated as a neotype since it is accompanied by a printed label with the note “G. Woronow. Notae criticae”, on which Jurij Nikolaewitch Woronow, the author of the name, handwrote “*Iris timofejewii* m. March (19)24”. It was collected in the vicinity of Khadzhalmakhi Village, Levashinsky Raion, Republic of Dagestan, Russia.

(iii) The name *I. astrachanica* was first validly published by Rodionenko in 1977 [15]. In the protologue of *I. astrachanica*, a single gathering was designated as the type. At least five undated specimens at LE (LE01015406–LE01015410!), accompanied by labels with the note “*Iris astrachanica* Rodion.”, handwritten by Rodionenko before 1977, belong to the original material of *I. astrachanica* and are syntypes (see Arts. 9.6 and 40, Note 1 of the ICN). These specimens were collected by Sergei Ivanovitsch Korshinsky, a Russian botanist, near the Volga River estuary in the vicinity of Astrakhan, Russia, in approximately 1880–1883. One of the syntypes (LE01015406) is designated here as the lectotype of *I. astrachanica*. It corresponds to the protologue of the name and contains a label on which Rodionenko handwrote “*Iris astrachanica* Rodion. 27 February 1958”, and at the bottom of the sheet, he handwrote in Russian “pyl’tsa s borodavchatoi ekzinoi” (translated = “pollen with a papillate exine”).

In the extended circumscription presented here, *I. scariosa* includes five synonyms. The information on all the names, with full nomenclature citations and the main findings on the distribution and habitat of *I. scariosa*, is provided below.

***Iris scariosa*** Willd. ex Link, Jahrb. Gewächsk. 1(3): 71, 1820 ≡ *I. pumila* var. *scariosa* (Willd. ex Link) Schmalh., Fl. Sredn. Jushn. Rossii 2: 470, 1897.—Protologue citation: [origin not specified].—Lectotype (designated by Sennikov et al. [72] (p. 32)): [Russia], s.loc., [fl.], s.d., [*P.S. Pallas*] *s.n*. Herb. Willdenow (B-W00959010, sub “*Iris scariosa* 1” det. L. Schlechtendal, et “*Iris aphylla* L.” det. F.W. Klatt).—https://herbarium.bgbm.org/object/BW00959010 (accessed on 24 July 2024).—Further original material: *Iris scariosa*, e Sibiria, [fl.], s.d., *Pallas s.n*. Herb. D.F.L. von Schlechtendal ex Willdenow Herbarium (HAL0109667, see https://hal.jacq.org/HAL0109667, accessed on 24 July 2024; the label was erroneously replaced by that the specimen HAL0109666, see https://hal.jacq.org/HAL0109666, accessed on 24 July 2024).

= *Iris glaucescens* Bunge, Fl. Altaic. [Ledebour] 1: 58, 1829.—Protologue citation: “Hab. in pratis inter Schamanaicha et Wydricha (L. [Ledebour]) et in apricis montosis ad fl. Irtysch et Buchtarma (M. [Meyer])”.—Lectotype (designated by Alexeeva [87] (p. 416)): [Kazakhstan, East Kazakhstan Region] *Iris glaucescens* mihi, inter Schamanaicha et Wydricha, [fl.], 26 April (1826), *Ledebour 56*, Herb. Ledebour (LE01017920!).—http://re.herbariumle.ru/01017920 (accessed on 24 July 2024).—Further original material: [Kazakhstan, East Kazakhstan Region] *Iris glaucescens* Bunge. Altai, in pratis inter Schamanaicha et Wydricha, nec non pr. Riddersk et frant fluv. Ulba, [fl.], 1926, *Ledebour 46* (LE01017921!, see http://re.herbariumle.ru/01017921, accessed on 24 July 2024); *Iris glaucescens* Bunge (sec. definit. Bungcana), Altay, legi in montosis prope fortalitium Buchtarmainsk orientem versus usque ad fluv. Kurshhum, nec non in montib. Arkat et Dshigilen, [fl.], April 1826, [*Meyer*] *46*, Herb. Meyer (LE01017922!, see http://re.herbariumle.ru/01017922, accessed on 24 July 2024).

= *Iris eulefeldi* Regel, Trudy Imp. S.-Peterburgsk. Bot. Sada 5(2): 633, 1878 ≡ *I. glaucescens* [var.] β *eulefeldi* (Regel) Maxim. in Regel, Descr. Pl. Nov. 7: 212, 1879 ≡ *I. scariosa* var. *eulefeldi* (Regel) Maxim., Bull. Acad. Imp. Sci. Saint-Pétersbourg sér. 3, 26: 534, 1880.—Protologue citation: “Habitat in montibus thianschanicis in angustiis fluvii Talki (A. Regel)”.—Lectotype (designated by Boltenkov [88] (p. 263)):—[Specimen from a cultivated plant], [Information printed]: Ex horto bot. Petropolitano; [Information handwritten by E. Regel]: *Iris eulefeldi* Rgl., [fl.], May (18)78, *s.coll. s.n.* (LE01064421!).—(see [88] (p. 264, f. 1)).—Further original material: [China, Xinjiang Uyghur Autonomous Region] *Iris glaucescens* Bge. var. *eulefeldi* Rgl. (sp[ecies]. pr[ovisorius].), bach Almaty nordwestl. u[rbs]. Kuldscha [Ghulja, or Yining County-level city, Ili Kazakh Autonomous Prefecture], 4–5000′, 26 May 1878 [fl.], *A. Regel s.n.* (BM01209592!, E00701613!, P02158900 [see http://coldb.mnhn.fr/catalognumber/mnhn/p/p02158900, accessed on 24 July 2024]); *Iris glaucescens* Bge. var. *eulefeldi* fl. Suidun [Shuiding, Huocheng County, Ili Kazakh Autonomous Prefecture], fl. intense azureo, 4–6000′, (25,26) April 1878, *A. Regel s.n.* (P02158904 [digital image!]), *Iris glaucescens* Bge. var. *eulefeldi* Rgl. (sp. pr.), Sarybulak, pr. Kuldscha, 4000′, [fl.], (10–17) April 1878, *A. Regel s.n.* (L01472127, see https://data.biodiversitydata.nl/naturalis/specimen/L.1472127, accessed on 24 July 2024).

= *Iris timofejewii* Woronow, Bot. Mater. Gerb. Glavn. Bot. Sada R.S.F.S.R. 5: 62, 1924, ***syn. nov*.**—Protologue citation: “Culta in sectione caucasica Horti Tiflisiensis e rhizomatibus a cl. A. Grossheim e Daghestaniae distr. Andi a. 1915 allatis”.—**Neotype** (designated here by E.V. Boltenkov) [Republic of Dagestan, Russia] Prov. Dagestan, distr Dargi, pr. p. Chodshal-makhi, in decliviis calcareis, 3100′, [fr.], 29 May 1901, [*F.N*.] *Alexeenko 2523* (LE01268154!).—http://re.herbariumle.ru/01268154 (accessed on 24 July 2024).

= *Iris astrachanica* Rodion., Dekorativ. Trav. Rast. Dlya Otkr. Grunta SSSR 1: 251, 1977.—Protologue citation: “In delta fl. Volga (viciniae opp. Astrachan) Korshinsky legit”.—**Lectotype** (designated here by E.V. Boltenkov): [Russia, Astrakhan Oblast] Astrakhan, [fl.], s.d., *S. Korzhinski s.n*. [originally in Russian] (LE01015406!).—http://re.herbariumle.ru/01015406 (accessed on 24 July 2024).—Further original material: [printed labels] Flora of the delta of the Volga River, vicinity of Astrakhan, [fl.], s.d., *S. Korshinsky s.n*. [originally in Russian] (LE01015407!, see http://re.herbariumle.ru/01015407, accessed on 24 July 2024); Flora of the delta of the Volga River, [fl.], s.d., *S. Korshinsky s.n*. [originally in Russian] (LE01015408–LE01015410, see http://re.herbariumle.ru/01015408, http://re.herbariumle.ru/01015409, and http://re.herbariumle.ru/01015410, accessed on 24 July 2024).

**Figure 8:**
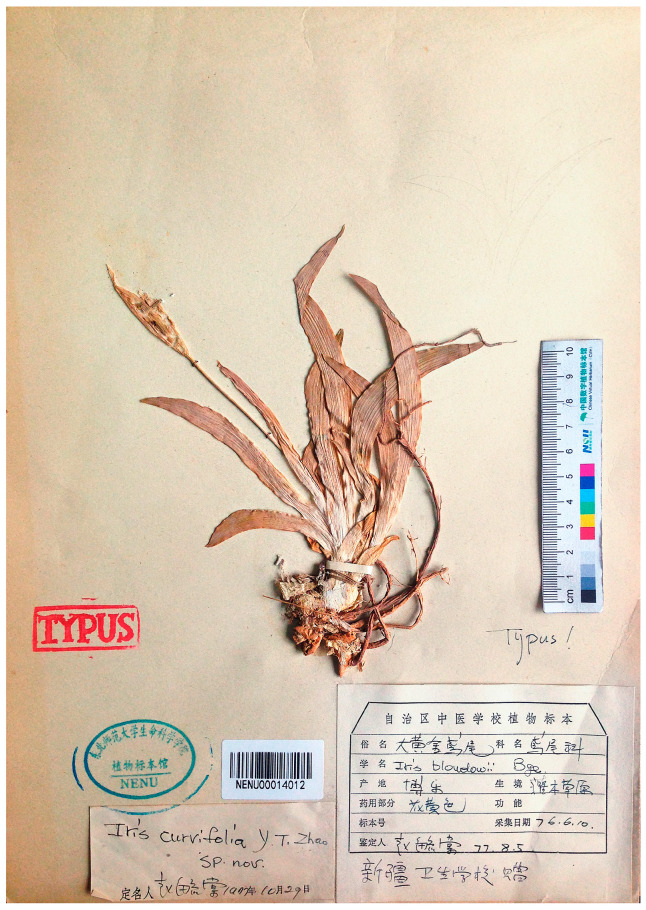
Holotype of *Iris curvifolia* (NENU00014012) (included with permission of the curator).

= *Iris curvifolia* Y.T.Zhao, Acta Phytotax. Sin. 20(1): 99, 1982, ***syn. nov*.**—Protologue citation: “Xinjiang: Bole, 10 June 1976. Y.F. Zhang (Typus in Herb. Univ. Normal. Bor.-Orient. Conservatur)”.—Holotype: [China] Xinjiang Uyghur Autonomous Region, Bortala, steppe, flower yellow, 10 June (19)76, *Y.F. Zhang s.n*. [originally in Chinese] (NENU00014012!).—Figure 8.—Paratypes (see Art. 9.7 of the ICN): Xinjiang Uyghur Autonomous Region, Tacheng Prefecture, Toli, 20 km north, steppe, flower yellow, 3 May 1974, *Compl. Exped. Xinjiang 7928* (NENU00014010!); Xinjiang Uyghur Autonomous Region, Altay Prefecture, Habahe County, Terekti, steppe, 1300 m, 26 June 1976, *s. coll. 10357* (NENU00014011!).

#### 4.1.2. Distribution and Ecology of *Iris scariosa*

In Russia, it is distributed in the eastern North Caucasus (western Republic of North Ossetia–Alania, northern Chechen Republic, and Republic of Dagestan), in the south of the European part (northeastern Stavropol Krai, southeastern Rostov Oblast, Republic of Kalmykia, Astrakhan Oblast, Volgograd Oblast, and southeastern Republic of Bashkortostan), and in the southern Western Siberia (Orenburg Oblast, in the south of the Chelyabinsk Oblast and Omsk Oblast, southwestern Novosibirsk Oblast, Altai Krai, and probably in the Altai Republic). Also, it occurs in Kazakhstan (Abai, Akmola, Aktobe, Almaty, East Kazakhstan, Jetisu, Karaganda, North Kazakhstan, Pavlodar, Ulytau, Kostanay, and West Kazakhstan regions) and China (western and northern Xinjiang Uygur Autonomous Region).

Krylov [13] noted that *I. scariosa* was distributed in the western and southern foothills of the Altai Mountains and was not distributed eastward. Meanwhile, we found a specimen at NSK (NSK0069117, sub *I*. *glaucescens*) collected in the north of Uymonskaya Steppe, Russia (“Altai, Ust-Koksinsky Raion, Terektinskiy mountain range, in the vicinity of Terekta Village, southern slope, stony steppe, 13 August 1984, *M. Lomonosova s.n*. [originally in Russian]”; see http://herb.csbg.nsc.ru:8081/, accessed on 24 July 2024). The record of *I. scariosa* from this locality was confirmed by the collector (M. Lomonosova, pers. comm.). This is probably the first record of *I. scariosa* from the Altai Republic, since neither it nor *I*. *glaucescens* are listed for this territory in the literature (e.g., [17,89]). Unfortunately, we do not know about any other collections of *I. scariosa* from this locality over the past 40 years. The finding of *I. scariosa* in the Altai Republic in May 2024 (P. Kosachev, pers. comm.) was not confirmed, and, therefore, needs further clarification.

According to references (sub *I*. *glaucescens*) [6,17,90], *I. scariosa* is found in Uvs Aimag, northwestern Mongolia. However, we did not encounter any herbarium specimens that would confirm the distribution of *I. scariosa* in Mongolia.

*Iris scariosa* can be found at elevations ranging from below sea level to 2700 m a.s.l. It occurs in stony, sandy, or gravelly habitats; on saline, clayey or limestone soils in dry steppes; grasslands on sunny hillsides, slopes, or terraces of low mountains; or beside ditches. The flowering period is from late April to mid-May, and the fruiting period is from July to August. Mature seeds have a sticky and sweet-tasting surface (A. Grebenjuk, pers. comm.) that, in our opinion, attracts ants and can be involved in the seed dispersal (i.e., myrmecochory).

### 4.2. Notes on the Iris subg. Iris Classification

In the recent classifications of *I*. subg. *Iris*, six sections were recognized: an autonymic section, *I*. sect. *Hexapogon*, *I*. sect. *Oncocyclus*, *I.* sect. *Psammiris*, *I.* sect. *Pseudoregelia*, and *I*. sect. *Regelia* [16,36]. Nonetheless, *I*. sect. *Hexapogon*, *I*. sect. *Regelia*, and *I*. sect. *Oncocyclus* are strongly supported as sister taxa by references [37,61,91]. Within the *I*. subg. *Iris* clade, we revealed four well-supported monophyletic groups (haplogroups in the MJ network and clusters in the phylogenetic tree). We suggested the taxonomic rank of these four groups to be the same and, therefore, we treated them at the sectional rank as follows: (1) *I*. sect. *Iris*, (2) *I*. sect. *Hexapogon*, (3) *I.* sect. *Psammiris*, and (4) *I.* sect. *Pseudoregelia*.

*Iris* sect. *Iris* is monophyletic with the type species *I. germanica* nesting in its clade (Figure 4). In this section, two subclades are resolved (Figure 4, see arrows) that do not correspond to frequently described subgroups such as *I*. ser. *Pumilae* (plants dwarf) and *I*. ser. *Elatae* G.H.M.Lawr. (plants medium to tall), as was reported in reference [37].

In *Iris*, the epithet *Hexapogon* was first used by Bunge [92] (p. 329) in the name of an unranked subdivision (Art. 37.1 of the ICN) of the genus, comprising *I. falcifolia* Bunge and *I. filifolia* Bunge (nom illeg., Art. 53.1 of the ICN), which are taxonomic synonyms of *I. longiscapa* [93]. Bunge noted that these plants had beards on both the inner and outer perianth segments as follows: “*laciniis perigonii omnibus barbatis*”. The taxon *Hexapogon* was assigned a sectional rank by Baker [94] as *I*. sect. *Hexapogon*, etc. (see below). Rodionenko [95] resurrected *I*. sect. *Hexapogon* that comprised bearded irises with arillate seeds in it. Subsequently, he combined all species of the genus *Iris* with seeds containing an aril into *I*. subg. *Arillosae* Rodion. [96], including the five sections of bearded irises according to references [16,36]. However, *I*. subg. *Arillosae* cannot be considered monophyletic [37]. According to the presented molecular data, *I.* sect. *Hexapogon* comprises species of three previously recognized sections, i.e., *I*. sect. *Hexapogon*, *I*. sect. *Oncocyclus*, and *I*. sect. *Regelia*, treated here at a serial rank. We believe that the main diagnostic feature of the *I.* sect. *Hexapogon* species is the presence of hairs on the adaxial side not only of the falls but also of the standards. In the species of *I*. ser. *Hexapogon* and *I*. ser. *Regelia*, the standards have a conspicuous, more or less linear beard of hairs down the claw, whereas in the *I*. ser. *Oncocyclus* species the standards have occasional hairs at the base of the claw.

#### 4.2.1. List of Taxa

The species of *I.* subg. *Iris* are distributed in the north temperate zone of Eurasia. The subgenus comprises four sections as circumscribed below. The composition of *I.* sect. *Hexapogon* is restored in the present study. It combines three groups, the autonymic series and two series proposed here, *I.* ser. *Oncocyclus* and *I.* ser. *Regelia*.

***Iris*** subg. ***Iris***

(I) ***Iris*** sect. ***Iris***.—Lectotype (designated by Britton and Brown [97] (p. 536)): *I. germanica* L.

It comprises over 20 species occurring mainly in Europe around the Mediterranean. It has long been recognized as a challenging group in which some species (e.g., *I. albicans* Lange, *I. kashmiriana* Baker, and *I. germanica*) are considered to be of hybrid origin, known only in cultivation or as naturalized plants escaped from cultivation.

(II) ***Iris*** sect. ***Hexapogon*** (Bunge) Baker, Gard. Chron., new. ser., 5: 527, 1876 ≡ *I*. [unranked] *Hexapogon* Bunge, Beitr. Fl. Russl.: 329, 1852 ≡ *I.* subg. *Hexapogon* (Bunge) Alef., Bot. Zeitung (Berlin) 21(40): 296, 1863, *nom. illeg. superfl.* (Art. 52.1 of the ICN) ≡ *I.* subg. *Hexapogon* (Bunge) Klatt, Linnaea 34: 592, 1866 ≡ *I.* subsect. *Hexapogon* (Bunge) Benth. in Benth. & Hook.f., Gen. Pl. 3(2): 687, 1883.—Lectotype (designated by Lawrence [60] (p. 354)): *Iris falcifolia* Bunge (a taxonomic synonym of *I. longiscapa* [93]).

(1) ***Iris*** ser. ***Hexapogon***

It is considered unispecific, including only *I. longiscapa* that is distributed mainly in desert and semi-desert areas of Central Asia (in Uzbekistan, Kazakhstan, and Turkmenistan, Tajikistan, and Afghanistan) and also in Iran and southwestern Pakistan [93]. *Iris longiscapa* shows a chromosome number of 2*n* = 18 [31,98], which is unique in *I*. subg. *Iris*.

(2) ***Iris*** ser. ***Regelia*** (Foster) Bolt., ***stat***. ***nov***. ≡ *I.* [unranked] *Regelia* Foster, Gard. Chron., ser. 3, 4: 36, 1888 ≡ *I.* subg. *Regelia* (Foster) Baker, Handb. Irid.: 20, 1892 ≡ *I.* sect. *Regelia* (Foster) Lynch, Book of the *Iris*: 116, 1904.—Lectotype (designated by Taylor [36] (415)]: *Iris korolkowii* Regel.

It comprises about six species occurring in Central Asia (southern Kazakhstan, Afghanistan, Tajikistan, Uzbekistan, and Turkmenistan). Two species, *I. stolonifera* and *I. hoogiana*, with 2*n* = 44, are amphidiploids, characterized by a wide-spreading rhizome bearing slender stolons, whereas the other species, with 2*n* = 22, are considered as diploids, characterized by a comparatively compact rhizome [99]. Among the latter species, *I. afghanica* is very characteristic, presumably holding a separate position (Figure 3 and Figure 4).

(3) ***Iris*** ser. ***Oncocyclus*** (Siemssen) Bolt., ***stat***. ***nov***. ≡ *Oncocyclus* Siemssen, Bot. Zeitung (Berlin) 4(41): 706, 1846 ≡ *Iris* subg. *Oncocyclus* (Siemssen) Alef., Bot. Zeitung (Berlin) 21(40): 296, 1863 ≡ *I.* sect. *Oncocyclus* (Siemssen) Baker, Gard. Chron., new. ser., 5: 527, 1876 ≡ *I.* subsect. *Oncocyclus* (Siemssen) Benth. et Hook.f., Gen. Pl. 3(2): 687, 1883.—Lectotype (designated by Lawrence [60] (p. 355)): *Iris paradoxa* Steven.

This is the broadest and very variable group in which many species have been described on the basis of weak morphological differences [100,101,102]. It comprises about 40 species, all with 2*n* = 20 [103], well adapted to arid conditions of the Middle East (Egypt, Israel, Palestine, Jordan, Lebanon, Syria, Turkey, Iraq, and Iran). They also grow in southern Turkmenistan, Transcaucasus, and in the Republic of Dagestan, Russia.

(III) ***Iris*** sect. ***Psammiris*** (Spach) J.J.Taylor, Proc. Biol. Soc. Washington 89(35): 417, 1976 ≡ *I*. subg. *Psammiris* Spach, Ann. Sci. Nat., Bot., ser. 3, 5(1): 110, 1846.—Holotype: *Iris arenaria* Waldst. et Kit. (a taxonomic synonym of *I. humilis* [39]).

Currently, the section has one of the best elaborated systematics [39]. It comprises only five species and is subdivided into an autonymic series (with *I. bloudowii*, *I. humilis*, and *I. vorobievii*) and two unispecific series: *I*. ser. *Potaninia* Doronkin with *I. potaninii* and *I*. ser. *Tigridiae* Doronkin with *I. tigridia*. They are distributed in steppes from southeastern Europe through southern Siberia, northern Kazakhstan, China, and Mongolia to the Russian Far East at elevations of 10–2800 m. *Iris humilis* is the most widely distributed and northernmost representatives of *I*. subg. *Iris*.

(IV) ***Iris*** sect. ***Pseudoregelia*** Dykes, Gen. *Iris*: 129, 1913 ≡ *I.* subsect. *Pseudoregelia* (Dykes) G.H.M. Lawr. 1953, Gentes Herb. 8(4): 356.—Lectotype (designated by Taylor [36] (419)]: *I. kemaonensis* Wall. ex Royle (“*I. kamaonensis* Wall. ex D.Don”).

It comprises about 10 species growing in mountains in China, Bhutan, northern India, northern Myanmar, Nepal, and Pakistan. Inhabiting elevations up to 5800 m, *I. thoroldii* is the highest-elevation species in the genus *Iris* and, possibly, in Iridaceae [104].

#### 4.2.2. The Key

The species of *I*. subg. *Iris* are rhizomatous perennials characterized by not swollen and not tuberlike adventitious roots, unifacial flat leaves, and flowers with a conspicuous beard on the falls. Below is a key to the *I*. subg. *Iris* taxa recognized in the present study.

1. Seed lack aril; falls with compact beard; rhizome stout, short-branched… *Iris* sect. *Iris*

– Seed arillate… 2

2. Aril much smaller than seed; standards lack hairs… 3

– Aril large, ring-shaped; standards with hairs… 4 (*Iris* sect. *Hexapogon*)

3. Aril conspicuous; rhizome compact, gnarled, not stoloniferous… *Iris* sect. *Pseudoregelia*

– Aril small, flat, disk-shaped; rhizome shortly creeping or stoloniferous… *Iris* sect. *Psammiris*

4. Standards with occasional hairs at base of claw; stem one-flowered, with flower usually very large… *Iris* ser. *Oncocyclus*

– Standards with conspicuous linear beard; stem more often two-flowered… 5

5. Stem leafless, with 3–4 bracts; rosette leaves narrow, <0.5 cm in width… *Iris* ser. *Hexapogon*

– Stem bears cauline leaves and two bracts; rosette leaves broader, >0.5 cm in width… *Iris* ser. *Regelia*

## 5. Conclusions

To consider the taxonomy of *I. scariosa* more in detail, we compared morphological characteristics and conducted molecular phylogenetic analyzes using sequence data for six chloroplast DNA regions. These are the most comprehensive phylogenetic analyses to date for the species. *Iris scariosa* is distinguished by its high variability in the morphological characters, especially in the flower color. We could not find any discontinuities of variation independent of environmental influences or any geographical pattern for these variations. Our major results are as follows: (1) the molecular data confirm that *I. glaucescens* is a synonym of *I. scariosa*; (2) the molecular data and the thorough examination of living plants confirm that *I. timofejewii*, recognized on the basis of morphology and considered as endemic to the Republic of Dagestan, Russia, is a synonym of *I. scariosa*; (3) a critical evaluation of the original material and literature have shown that *I. curvifolia* and *I. scariosa* are the same taxon; and (4) the exine ornamentation in *I. scariosa* is of clavate–baculate–granulate type. As a consequence, our findings have clarified the composition of *I*. subg. *Iris* in Russia, where it is represented by nine species. Currently, these include five species of *I*. sect. *Psammiris* [39], *I. acutiloba*, and also three species of *I*. sect. *Iris*. Of the latter, *I. aphylla* and *I. pumila* are distributed in the European part of Russia and the North Caucasus, while *I. scariosa* grows in the south of the European part of Russia, in the east of the North Caucasus, and in the south of the Western Siberia.

This study also offers a path forward to a revised infrageneric classification of the genus *Iris* based on molecular data. All species presented in this study are divided into three clades that correspond to three subgenera: *I.* subg. *Iris*, *I.* subg. *Pardanthopsis*, and *I.* subg. *Limniris*. The sister clade to *I*. subg. *Iris* is *I.* subg. *Pardanthopsis* with *I. dichotoma* and *I. domestica*, which should be treated as legitimate species of the genus *Iris* (also see [37,61,63,64]). As the first step, the classification of *I*. subg. *Iris* is revised here. The data that we present have several important implications for the taxonomy of *I.* subg. *Iris*: (1) four monophyletic clades correspond to the sections, i.e., *I.* sect. *Iris*, *I.* sect. *Hexapogon*, *I.* sect. *Psammiris*, and *I.* sect. *Pseudoregelia*, which are morphologically quite clearly distinguished; (2) *I.* sect. *Hexapogon* comprises three series, i.e., *I*. ser. *Hexapogon*, *I*. ser. *Oncocyclus*, and *I*. ser. *Regelia*, previously recognized as sections; and (3) our analyses confirm the split of monophyletic *I*. sect. *Iris* into two groups, but no pronounced morphological differences or geographic patterns have been found to explain this division. We believe that this classification provides a foundation for future endeavors. Nevertheless, more phylogenetic analyses within the genus are required. Also, further revision of the current subsectional system in *I*. sect. *Iris* and its taxonomic composition, as well as of *I*. ser. *Oncocyclus*, *I*. sect. *Pseudoregelia*, and *I*. ser. *Regelia*, needs substantial efforts.

## Figures and Tables

**Table 1:** Sampled *Iris* taxa with voucher information and GenBank accession numbers.

Code/Species (Haplotype)	Locality (Voucher *)	Coordinates: °N, °E	GenBank Accession Nos. *trnH-psbA*/*rps4-trnS*/*trnS-trnG*/ *trnL-trnF*/*ndhF*/*ycf1*
*Iris* subg. *Iris*			
*I.* sect. *Iris*			
*I. timofejewii* Woronow			
S1 (H1)	Russia, Dagestan, vicinity of Botlikh Village, *Boltenkov 178* (VBGI)	42.693889, 46.249444	PP724851/PP724882/PP724913/PP724944/PP724975/PP725005
S2 (H2)	Russia, Dagestan, vicinity of Muni Village, *Boltenkov 179* (VBGI)	42.676667, 46.287222	PP724852/PP724883/PP724914/PP724945/PP724976/PP725006
S3 (H3)	Russia, Dagestan, vicinity of Usukhchai Village, *Boltenkov 176* (VBGI)	41.430278, 47.938611	PP724853/PP724884/PP724915/PP724946/PP724977/PP725007
S4 (H4)	Russia, Dagestan, near Sagrinskiy Most, *Boltenkov 180* (VBGI)	42.749722, 46.626944	PP724854/PP724885/PP724916/PP724947/PP724978/PP725008
S5 (H5)	Russia, Dagestan, vicinity of Tsudakhar Village, *Boltenkov s.n.* (VBGI)	42.328889, 47.163056	PP724857/PP724888/PP724919/PP724950/PP724981/PP725011
S6 (H5)	Russia, Dagestan, Gunib Village, *Boltenkov s.n.* (VBGI)	42.390000, 46.959444	PP724856/PP724887/PP724918/PP724949/PP724980/PP725010
S7 (H5)	Russia, Dagestan, vicinity of Khkem Village, *Boltenkov 186* (VBGI)	41.473889, 47.756944	PP724855/PP724886/PP724917/PP724948/PP724979/PP725009
*I. scariosa* Willd. ex Link			
S8 (H6)	Russia, vicinity of Astrakhan, *S. Zenin s.n.* (cult.)	46.392341, 47.956134	PP724858/PP724889/PP724920/PP724951/PP724982/PP725012
*I. glaucescens* Willd. ex Link			
S9 (H8)	Kazakhstan, Akmola Region, vicinity of Lesnoye, *P. Kosachev* et al. *s.n*. (VBGI)	52.584420, 68.621720	PP724859/PP724890/PP724921/PP724952/PP724983/PP725013
S10 (H10)	Kazakhstan, Akmola Region, vicinity of Aksu Village, *P. Kosachev* et al. *s.n*. (VBGI)	52.479800, 71.941320	PP724860/PP724891/PP724922/PP724953/PP724984/PP725014
S11 (H8)	Kazakhstan, Karaganda Region, vicinity of Batyk Village, *P. Gudkova & E. Kriuchkova s.n.* (VBGI)	48.896389, 72.395556	PP724861/PP724892/PP724923/PP724954/PP724985/PP725015
S12 (H9)	Kazakhstan, Karaganda Region, south of Aksu-Ayuly Village, *P. Gudkova & E. Kriuchkova s.n.* (VBGI)	48.616944, 73.700833	PP724862/PP724893/PP724924/PP724955/PP724986/PP725016
S13 (H7)	Kazakhstan, Pavlodar Region, 1 km western of Sherbakty Village, *P. Kosachev* et al. *s.n*. (VBGI)	52.473216, 78.098317	PP724863/PP724894/PP724925/PP724956/PP724987/PP725017
S14 (H10)	Kazakhstan, East Kazakhstan Region, vicinity of Ust-Kamenogorsk, *M. Koldaeva s.n*. (VBGI, cult.)	50.012073, 82.431543	PP724864/PP724895/PP724926/PP724957/PP724988/PP725018
S15 (H11)	Russia, Altai Krai, vicinity of Lake Kuchuk, *A. Grebenjuk s.n*. (LE)	52.616234, 80.100987	PP724865/PP724896/PP724927/PP724958/PP724989/PP725019
*I. aphylla* L.			
A1	Russia, Stavropol Krai, Urochishche Russkaya Lesnaya Dacha, *Boltenkov 143* (VBGI)	45.101111, 41.886389	PP724866/PP724897/PP724928/PP724959/PP724990/PP725020
A2	Russia, Dagestan, Mikrakh Village, *Boltenkov 175* (VBGI)	41.369167, 47.892500	PP724867/PP724898/PP724929/PP724960/PP724991/PP725021
A3	Russia, Stavropol Krai, vicinity of Yutsa Village, *Boltenkov 157* (VBGI)	43.962222, 43.024722	PP724869/PP724900/PP724931/PP724962/PP724993/PP725023
A4	Russia, Stavropol Krai, Mount Mashuk, *Boltenkov 166* (VBGI)	44.038889, 43.089722	PP724868/PP724899/PP724930/PP724961/PP724992/PP725022
*I. pumila* L.			
P1	Russia, Stavropol Krai, Sengileyevskoye Vodokhranilishche, *Boltenkov 149* (VBGI)	45.009722, 41.803333	PP724871/PP724902/PP724933/PP724964/PP724995/PP725025
P2	Russia, Rostov Oblast, vicinity of Lysogorka Village, *Boltenkov 150* (VBGI)	47.717778, 39.210278	PP724870/PP724901/PP724932/PP724963/PP724994/PP725024
*I. alberti* Regel	Kyrgyzstan, Chychkan River gorge, *Boltenkov 132* (VBGI)	42.076389, 72.813056	PP724873/PP724904/PP724935/PP724966/PP724997/PP725027
*I. imbricata* Lindl.	Armenia, between Aghitu and Vaghatin villages, *M. Oganesian* et al. *114* (LE)	39.509722, 46.097778	PP724874/PP724905/PP724936/PP724967
*I. reichenbachii* Heuff.	Bulgaria, near Ponor Pass, *A. Erst & A. Tashev s.n*. (VBGI)	42.911758, 23.163012	PP724872/PP724903/PP724934/PP724965/PP724996/PP725026
*I.* sect. *Oncocyclus* (Siemssen) Baker *I. iberica* M.Bieb.			
Ar1	Armenia, vicinity of Nor Artamet Village, *Boltenkov s.n*. (VBGI)	40.291111, 44.547778	PP724877/PP724908/PP724939/PP724970/PP725000/PP725030
Ar2	Armenia, vicinity of Garni Village, *Boltenkov s.n*. (VBGI)	40.122778, 44.695556	PP724878/PP724909/PP724940/PP724971/PP725001/PP725031
*I. acutiloba* C.A.Mey.	Russia, Dagestan, vicinity of Novyy Deybuk Village, *Boltenkov 182* (VBGI)	42.402500, 47.938333	PP724881/PP724912/PP724943/PP724974/PP725004/PP725034
*I.* sect. *Hexapogon* (Bunge) Baker			
*I. longiscapa* Ledeb.			
Uz1	Uzbekistan, Surxondaryo Region, vicinity of Termez, *O. Turginov s.n*. (TASH)	37.383333, 67.366667	PP724879/PP724910/PP724941/PP724972/PP725002/PP725032
Uz2	Uzbekistan, Surxondaryo Region, vicinity of Aqtash Village, *O. Turginov s.n*. (TASH)	37.561182, 66.670855	PP724880/PP724911/PP724942/PP724973/PP725003/PP725033
*I.* sect. *Regelia* Lynch			
*I. stolonifera* Maxim.	Tajikistan, Choltosh Village, *P. Gudkova s.n.* (VBGI)	38.571389, 68.493611	PP724876/PP724907/PP724938/PP724969/PP724999/PP725029
*I. korolkowii* Regel	Uzbekistan, Namangan Region, Pop District, *M. Turgunov s.n*. (TASH, cult.)	–	PP724875/PP724906/PP724937/PP724968/PP724998/PP725028
*I.* sect. *Psammiris* (Spach) J.J.Taylor			
*I. potaninii* Maxim.	Russia, Zabaykalsky Krai, Lake Zun-Torey, *Boltenkov 77* (VBGI)	50.12972, 115.70361	*ON569466/ON569554/ON569642/ON569730/*PP725035/PP739309
*I. vorobievii* N.S.Pavlova	Russia, Primorsky Krai, Kraskino, *Boltenkov s.n*. (VBGI)	42.725, 130.93361	*ON569514/ON569602/ON569690/ON569778/*PP725036/PP739310
*I. bloudowii* Ledeb.	Kazakhstan, Almaty Region, west of Qapal, *A. Grebenjuk 161* (LE)	45.02486, 78.94919	*ON569476/ON569564/ON569652/ON569740/*PP725037/PP739311
*I. humilis* Georgi	Russia, Buryatia, Mount Spyashchiy Lev, *Boltenkov 63* (VBGI)	51.53833, 107.34611	*ON569491/ON569579/ON569667/ON569755/*PP725038/PP739312
*I. tigridia* Bunge	Russia, Zabaykalsky Krai, Aginsky District, Lake Khaptsagaytuy, *Boltenkov 72* (VBGI)	50.6167, 114.88777	*ON569520/ON569608/ON569696/ON569784/*PP725039/PP739313
*I.* sect. *Pseudoregelia* Dykes			
*I. thoroldii* Baker	China, Qinghai, northern slope of Jiangluling, *D.G. Long* et al. *148* (E00141064)	35.56576, 99.98481	*ON569530/ON569618/ON569706/ON569794*
*I. goniocarpa* Baker	China, Gansu, Wanmaoxiang, *SQAE 85* (E)	34.8013, 103.20255	*ON569527/ON569615/ON569703/ON569791*
Outgroup accessions			
*I.* subg. *Pardanthopsis* (Hance) Baker			
*I. dichotoma* Pall.	Russia, Amur Oblast, *M. Baranova s.n*. (cult.)	–	*LT978555/LT981297/LT984447/LT984483*
*I.* subg. *Limniris* (Tausch) Spach			
*I.* ser. *Lacteae* Doronkin			
*I. lactea* Pall.	Russia, Zabaykalsky Krai, Kharanor, *Chernova s.n*. (IRK)	–	*LT627854/LN871708/LN871662/LN871625/*PP725040/PP739314
*I. oxypetala* Bunge	China, Shaanxi, Suyde, *Kabanov s.n*. (LE)	–	*LT627844/LT627950/LT627975/LT627911/*PP725041/PP739315
*I. tibetica* (Dykes) Bolt.	China, Qinghai, Xining to Ta Er, *Long* et al. *3* (E)	–	*LT627893/LT627939/LT627998/LT627933/*PP725042/PP739316
*I.* ser. *Laevigatae* (Diels) G.H.M.Lawr.			
*I. ensata* Thunb.	Russia, Primorsky Krai, Zarubino, *Boltenkov s.n*. (VBGI)	–	*LT627896/LT628012/LT628022/LT628002*
*I. laevigata* Fisch.	Russia, Primorsky Krai, Roshchino, *Pshennikova s.n*. (VBGI)	–	*LT627897/LT628013/LT628024/LT628003*
*I. pseudacorus* L.	Russia, Vladivostok, *Boltenkov s.n*. (VBGI, cult.)	–	*LT627898/LT628014/LT628025/LT628004*
*I*. ser. *Ruthenicae* (Diels) G.H.M.Lawr.			
*I. uniflora* Pall. ex Link	Russia, Primorsky Krai, Zarubino, *Boltenkov s.n.* (VBGI)	–	*LT627902/LT628018/LT628029/LT628008*
*I.* ser. *Sibiricae* (Diels) G.H.M.Lawr.			
*I. sibirica* L.	Russia, Udmurt Republic, Perevoznoye, *Melnikov s.n.* (VBGI)	–	*LT978537/LT981279/LT984429/LT984462/*PP725047/PP739321
*I. bulleyana* Dykes	China, Yunnan, Zhongdian, *M.G. Pimenov* et al. *432* (MW0735232)	–	*LT627895/LT628011/LT628021/LT628001/*PP725043/PP739317
*I. delavayi* Micheli	China, Yunnan, Dali Xian, Yinglofen, *Sino-Amer. Bot. Expedition 959* (MHA)	–	*LT978552/LT981294/LT984444/LT984477/*PP725044/PP739318
*I. clarkei* Baker ex Hook.f.	India, Ladakh, Kargil, *C.A. Chadwell 82* (E, cult.)	–	*LT978547/LT981289/LT984439/LT984472/*PP725045/PP739319
*I. wilsonii* C.H.Wright	China, Yunnan, Little Zhongdian, *E.J. Cowley 566* (Kew no. 1990-3457, cult.)	–	*LR597339/LR597355/LR597371/LR597387/*PP725046/PP739320

* Herbarium codes are according to reference [38]. A dash (“–”) indicates that data were not provided. The accession numbers highlighted in italics are reported in references [39,40,41,42]. Cult., cultivated.

**Table 2:** Nucleotide divergence between species within *Iris* sect. *Iris*: below the diagonal, average number of nucleotide substitutions per site (*D_XY_*); above the diagonal, average number of nucleotide differences (in brackets, the number of fixed differences).

Species	*I. timofejewii*	*I. scariosa*	*I. glaucescens*	*I. aphylla*	*I. reichenbachii*	*I. imbricata*	*I. pumila*	*I. lutescens*	*I. alberti*	*I. germanica*
*I. timofejewii*	–	3.571 (1)	6.878 (2)	15.714 (12)	14.571 (11)	11.571 (8)	16.857 (10)	12.571 (9)	7.143 (5)	19.571 (16)
*I. scariosa*	0.00052	–	5.714 (3)	14.500 (13)	18.000 (18)	15.000 (15)	16.000 (11)	16.000 (16)	12.000 (12)	23.000 (23)
*I. glaucescens*	0.00099	0.00082	–	17.786 (13)	19.714 (13)	16.714 (10)	18.857 (10)	17.429 (10)	13.714 (7)	24.714 (18)
*I. aphylla*	0.00227	0.00209	0.00257	–	14.750 (9)	21.750 (16)	22.250 (16)	20.750 (15)	20.750 (15)	19.750 (14)
*I. reichenbachii*	0.00210	0.00260	0.00284	0.00213	–	15.000 (15)	22.000 (17)	16.000 (16)	14.000 (14)	13.000 (13)
*I. imbricata*	0.00146	0.00216	0.00241	0.00314	0.00216	–	17.000 (12)	11.000 (11)	11.000 (11)	20.000 (20)
*I. pumila*	0.00243	0.00231	0.00272	0.00321	0.00317	0.00245	–	5.500 (3)	18.000 (13)	27.000 (22)
*I. lutescens*	0.00181	0.00231	0.00251	0.00299	0.00231	0.00159	0.00079	–	12.000 (12)	21.000 (21)
*I. alberti*	0.00103	0.00173	0.00198	0.00299	0.00202	0.00159	0.00260	0.00173	–	19.000 (19)
*I. germanica*	0.00282	0.00332	0.00356	0.00285	0.00187	0.00288	0.00389	0.00303	0.00274	–

**Table 3:** Nucleotide divergence between sections within *Iris* subg. *Iris*: below the diagonal, average number of nucleotide substitutions per site (*D_XY_*); above the diagonal, average number of nucleotide differences (in brackets, the number of fixed differences).

Section	*Psammiris*	*Pseudoregelia*	*Iris*	*Oncocyclus*	*Hexapogon*	*Regelia*
*Psammiris*	–	98.533 (63)	69.685 (46)	78.686 (60)	0.0083.300 (67)	78.450 (50)
*Pseudoregelia*	0.01470	–	86.462 (55)	103.905 (79)	108.167 (84)	101.167 (69)
*Iris*	0.01010	0.01295	–	67.401 (50)	69.846 (54)	65.683 (41)
*Oncocyclus*	0.01136	0.01551	0.00977	–	12.571 (6)	29.357 (12)
*Hexapogon*	0.01203	0.01614	0.01013	0.00181	–	32.250 (15)
*Regelia*	0.01133	0.01510	0.00952	0.00423	0.00465	–

**Table 4:** Morphological comparison of *Iris scariosa* with *I. timofejewii*.

No.	Characters	*I. scariosa*	*I. timofejewii*
1	Rhizome shape	Thick, tough, creeping	Thick, tough, creeping
2	Rhizome diameter	0.6–3	0.6–2
3	Rosette leaf shape	Ensiform, falcate or straight	Ensiform, falcate or straight
4	Rosette leaf texture	Chartaceous	Chartaceous
5	Rosette leaf apex shape	Narrowly acute or acute	Narrowly acute
6	Rosette leaf surface	Glaucous	Glaucous
7	Rosette leaf length	6–34	5–25
8	Rosette leaf width	0.3–2.2	0.3–1.5
9	Stem height	2–25	1–15
10	Number of cauline leaves	1	1
11	Cauline leaf length	5.5–12.8	5–9
12	Number of bracts	2	2
13	Number of bracteoles	1	1
14	Bract shape	Broadly-lanceolate	Broadly-lanceolate
15	Bract texture	Membranous	Membranous
16	Bract length	3–8.5	3.2–6
17	Pedicel length	0.1–0.7	0.1–0.6
18	Perianth tube length	2.2–5.5	2.2–4.5
19	Number of flowers	1–2	1–2
20	Flower color	Very variable	Variable
21	Flower diameter	3.5–5	3.5–5
22	Fall shape	Obovate	Obovate
23	Standard shape	Oblanceolate	Oblanceolate
24	Fruit shape	Oblong-ellipsoid	Oblong-ellipsoid
25	Fruit texture	Coriaceous	Coriaceous
26	Fruit length	2.5–8	2–5
27	Fruit diameter	1.5–3	1.2–1.7
28	Seed shape	Pyriform	Pyriform
29	Seed color	Reddish brown	Reddish brown n
30	Seed length	4.5–8 mm	5.4–7.6 mm
31	Seed diameter	2.9–5.5 mm	3.3–5.3 mm

All measurements are in centimeters, except for seeds. Data are presented as range (minimum and maximum values). See supplementary raw data in Table S2 for more details; for illustrations, see Figure 1, Figure 5 and Figure 6.

## Data Availability

The sequences resulting from this study are available in the GenBank database (https://www.ncbi.nlm.nih.gov/, accessed on 24 July 2024) with the accession numbers PP724851–PP725047 and PP739309–PP739321.

## Supplementary Materials

The following supporting information can be downloaded at: https://www.mdpi.com/article/10.3390/plants13172349/s1, Table S1: GenBank accession numbers for the *Iris* species sequences used in the study (see https://www.ncbi.nlm.nih.gov/, accessed on 15 March 2024); Table S2: Raw data of the morphological analysis of the *Iris* species studied (the numbers of the morphological characters correspond to those in Table 4).

## References

[1] Link H.F. (1820). Der botanische garten bei Berlin und die Willdenowsche kräutersammlung. Jahrb. Gewächsk..

[2] Fedtschenko B.A., Komarov V.L. (1935). Iris. Flora of the USSR.

[3] Waddick J.W., Zhao Y.-T. (1992). Iris of China.

[4] Tscherneva O.V., Kovalevskaja S.S. (1971). *Iris* L.. Conspectus Florae Asiae Mediae.

[5] Grubov V.I., Grubov V.I., Egorova T.V. (1977). Iridaceae. Plantae Asiae Centralis.

[6] Shevchenko G.T., Galushko A.I. (1979). Iris scariosa Willd. ex Link in the North Caucasus. Flora of the North Caucasus and Questions of its History.

[7] Tzvelev N.N., Fedorov A.A. (1979). Iridaceae. Flora Yevropeyskoy Chasti SSSR [Flora of the European Part of the USSR].

[8] Ledebour C.F. (1829). Flora Altaica.

[9] Maximowicz C.J. (1880). Diagnoses plantarum novarum asiaticarum, III. Bull. Acad. Imp. Sci. Saint-Pétersbourg.

[10] Baker J.G. (1892). Handbook of the Irideae.

[11] Dykes W.R. (1924). A Handbook of Garden Irises.

[12] Sand W.W.A. (1926). A study of Pogoniris varieties. Mem. N. Y. Agric. Exp. Stn..

[13] Krylov P.N., Krylov P.N. (1929). Iridaceae. Flora Zapadnoi Sibiri [Flora of the Western Siberia].

[14] Peckham E.A.S. (1939). Alphabetical Iris Check List.

[15] Rodionenko G.I., Avrorin N.A. (1977). Iris. Dekorativnye Travyanistye Rasteniya Dlya Otkrytogo Grunta SSSR.

[16] Mathew B. (1989). The Iris.

[17] Doronkin V.M., Malyshev L.I., Peschkova G.A. (1987). Iridaceae. Flora of Siberia.

[18] Service N., The Species Group of the British Iris Society (2012). Section *Iris*. A Guide to Species Irises: Their Identification and Cultivation.

[19] Catalogue of Life. https://www.catalogueoflife.org/.

[20] Encyclopedia of Life. https://eol.org/pages/42430800.

[21] Hortipedia. https://en.hortipedia.com/Main_page.

[22] iNaturalist. https://www.inaturalist.org.

[23] Plantarium. https://www.plantarium.ru/lang/en.html.

[24] Plants of the World Online. https://powo.science.kew.org/.

[25] SIGNA. http://www.signa.org/index.pl?Database.

[26] The American Iris Society Iris Encyclopedia. https://wiki.irises.org/Ird/IrdIris.

[27] The Global Biodiversity Information Facility. https://www.gbif.org/.

[28] Tropicos. https://tropicos.org/name/Search.

[29] World Flora Online. https://wfoplantlist.org/plant-list.

[30] World Plants. https://www.worldplants.de/world-plants-complete-list/complete-plant-list.

[31] Randolph L.F., Mitra J. (1961). Karyotypes of *Iris* species indigenous to the USSR. Am. J. Bot..

[32] Zakhariyeva O.I., Makushenko L.M. (1969). Chromosome numbers of monocotyledons belonging to the families Liliaceae, Iridaceae, Amaryllidaceae and Araceae. Bot. Zhurn..

[33] Murtazaliev R.A., Magomedov M.A. (2017). *Iris timofejewii*: Ecology, biology, introduction. Bot. Her. North Cauc..

[34] Solomon J., Shulkina T., Schatz G.E. (2014). Red list of the endemic plants of the Caucasus: Armenia, Azerbaijan, Georgia, Iran, Russia, and Turkey. Monogr. Syst. Bot. Mo. Bot. Gard..

[35] (2014). Iris timofejewii.

[36] Taylor J.J. (1976). A reclassification of *Iris* species bearing arillate seeds. Proc. Biol. Soc. Wash..

[37] Wilson C.A. (2017). Sectional relationships in the Eurasian bearded *Iris* (subgen. *Iris*) based on phylogenetic analyses of sequence data. Syst. Bot..

[38] Index Herbariorum. https://sweetgum.nybg.org/ih/.

[39] Boltenkov E.V., Artyukova E.V. (2023). New approach to the systematics of the section *Psammiris* (*Iris*, Iridaceae): What does chloroplast DNA sequence tell us?. Plants.

[40] Boltenkov E.V., Artyukova E.V., Kozyrenko M.M., Trias-Blasi A. (2018). *Iris tibetica*, a new combination in *I*. ser. Lacteae (Iridaceae) from China: Evidence from morphological and chloroplast DNA analyses. Phytotaxa.

[41] Boltenkov E., Artyukova E., Kozyrenko M., Erst A., Trias-Blasi A. (2020). *Iris sanguinea* is conspecific with *I. sibirica* (Iridaceae) according to morphology and plastid DNA sequence data. PeerJ.

[42] Boltenkov E.V., Artyukova E.V., Trias-Blasi A. (2021). Taxonomic composition of *Iris* subser. Chrysographes (Iridaceae) inferred from chloroplast DNA and morphological analyses. Plants.

[43] Woronow G. (1924). Diagnoses plantarum novarum praesertim e sectione caucasica Horti Tiflisiensis. Bot. Mater. Gerb. Glavn. Bot. Sada RSFSR.

[44] Kozyrenko M.M., Artyukova E.V., Boltenkov E.V., Lauve L.S. (2004). Somaclonal variability of *Iris pseudacorus* L. according to RAPD and cytogenetic analyses. Biotechnol. Russ..

[45] Kozyrenko M.M., Artyukova E.V., Zhuravlev Y.N. (2009). Independent species status of *Iris vorobievii* N.S.Pavlova, *Iris mandshurica* Maxim., and *Iris humilis* Georgi (Iridaceae): Evidence from the nuclear and chloroplast genomes. Russ. J. Genet..

[46] Wilson C.A. (2011). Subgeneric classification in *Iris* re-examined using chloroplast sequence data. Taxon.

[47] Wilson C.A., Padiernos J., Sapir Y. (2016). The royal irises (*Iris* subg. Iris sect. Oncocyclus): Plastid and low-copy nuclear data contribute to an understanding of their phylogenetic relationships. Taxon.

[48] Wilson C.A. (2014). The complete plastid genome sequence of *Iris gatesii* (Section *Oncocyclus*), a bearded species from southeastern Turkey. Aliso.

[49] Bonfield J.K., Smith K.F., Staden R. (1995). A new DNA sequence assembly program. Nucleic Acids Res..

[50] Gouy M., Guindon S., Gascuel O. (2010). SeaView version 4: A multiplatform graphical user interface for sequence alignment and phylogenetic tree building. Mol. Biol. Evol..

[51] Librado P., Rozas J. (2009). DnaSP v5: A software for comprehensive analysis of DNA polymorphism data. Bioinformatics.

[52] Bandelt H.-J., Forster P., Röhl A. (1999). Median-joining networks for inferring intraspecific phylogenies. Mol. Biol. Evol..

[53] Swofford D.L. (2002). PAUP*: Phylogenetic Analysis Using Parsimony (*and Other Methods), Version 4.0 b10.

[54] Ronquist F., Huelsenbeck J.P. (2003). MrBayes 3: Bayesian phylogenetic inference under mixed models. Bioinformatics.

[55] Miller M.A., Pfeiffer W., Schwartz T. Creating the CIPRES Science Gateway for inference of large phylogenetic trees. Proceedings of the Gateway Computing Environments Workshop (GCE 2010).

[56] Posada D., Crandall K.A. (1998). MODELTEST: Testing the model of DNA substitution. Bioinformatics.

[57] Chinese Virtual Herbarium. https://www.cvh.ac.cn/index.php.

[58] Beentje H. (2012). The Kew Plant Clossary.

[59] Punt W., Hoen P.P., Blackmore S., Nilsson S., Le Thomas A. (2007). Glossary of pollen and spore terminology. Rev. Palaeobot. Palynol..

[60] Lawrence G.H.M. (1953). A reclassification of the genus *Iris*. Gentes Herbarum.

[61] Tillie N., Chase M.W., Hall T. (2000). Molecular studies in the genus *Iris* L.: A preliminary study. Ann. Bot. (Roma) New Ser..

[62] Zhao Y.-T., Noltie H.J., Mathew B., Wu Z.-Y., Raven P.H. (2000). Iridaceae. Flora of China.

[63] Reeves G., Chase M.W., Goldblatt P., Rudall P., Fay M.F., Cox A.V., LeJeune B., Souza-Chies T. (2001). Molecular systematics of Iridaceae: Evidence from four plastid DNA regions. Am. J. Bot..

[64] Goldblatt P., Rodriguez A., Powell M.P., Davies T.J., Manning J.C., Van der Bank M., Savolainen V. (2008). Iridaceae ‘out of Australia’? Phylogeny, biogeography, and divergence time based on plastid DNA sequences. Syst. Bot..

[65] Wróblewska A., Brzosko E., Chudzińska E., Bordács S., Prokopiv A.I. (2010). Cytotype distribution and colonization history of the steppe plant *Iris aphylla*. Ann. Bot. Fenn..

[66] Turland N.J., Wiersema J.H., Barrie F.R., Greuter W., Hawksworth D.L., Herendeen P.S., Knapp S., Kusber W.-H., Li D.-Z., Marhold K. (2018). International Code of Nomenclature for Algae, Fungi, and Plants (Shenzhen Code) Adopted by the Nineteenth International Botanical Congress Shenzhen, China, July 2017 [Regnum Vegetabile Volume 159].

[67] The Plant Photo Bank of China. https://ppbc.iplant.cn/.

[68] Zhao Y.-T. (1982). New taxa of *Iris* L. from China. Acta Phytotax. Sin..

[69] Liu Y.-H. (1985). Flora in Desertis Reipublicae Populorum Sinarum.

[70] Fu L., Hong T. (2002). Higher Plants of China.

[71] Wu Z.-Y., Raven P.H. (2002). Flora of China Illustrations.

[72] Sennikov A., Khassanov F., Ortikov E., Kurbonaliyeva M., Tojibaev K.S. (2023). The genus *Iris* L. s. l. (Iridaceae) in the mountains of Central Asia biodiversity hotspot. Plant Divers. Cent. Asia.

[73] Jones A.G., Hiepko P. (1981). The Genus *Aster* s. l. (Asteraceae) in the Willdenow Herbarium at Berlin. Willdenowia.

[74] Pallas P.S. (1771). Reise Durch Verschiedene Provinzen des Russischen Reichs.

[75] Saksonov S.V., Senator S.A. (2012). Guide the Samara Flora 1851–2011.

[76] Dykes W.R. (1913). The Genus Iris.

[77] Pallas P.S. (1799). Bemerkungen auf Einer Reise in die Südlichen Statthalterschaften des Russischen Reichs in den Jahren 1793 und 1794.

[78] Rodionenko G.I. (1996). The rare *Iris*. Bull. Am. Iris Soc..

[79] Pinar N.M., Dönmez E.O. (2000). Pollen morphology of Turkish *Iris* L. (Iridaceae) with reference to evolutionary trends at the infrageneric level. Isr. J. Plant Sci..

[80] Mitić B., Halbritter H., Šoštarić R. (2013). Pollen morphology of the genus *Iris* L. (Iridaceae) from Croatia and surrounding area: Taxonomic and phylogenetic implications. Plant Syst. Evol..

[81] Hayrapetyan A.M., Muradyan A.H. (2022). Features of pollen morphology of species of the subgenus *Iris* (*Iris* L., Iridaceae) of the flora of Armenia. Biol. Zhurn. Armenii.

[82] Qian Y., Zhang H., Wu Z., Wang Z. (2011). Vegetation composition and distribution on the northern slope of Karlik Mountain to Naomaohu basin, East Tian shan Mountains. J. Arid Land.

[83] Catalogue of Life China. http://www.sp2000.org.cn/search/search_for_scientific_names.

[84] Subject Database of China Plant. http://www.plant.csdb.cn/.

[85] Boltenkov E.V. (2018). Taxonomic notes on *Iris* ser. *Lacteae* (Iridaceae) with typifications of fifteen names and one new combination. Phytotaxa.

[86] Boltenkov E.V., Menshakova M.Y., Gainanova R.I., Rumjantseva Z.Y. (2020). The first record of *Iris setosa* (Iridaceae) in Europe. Phytotaxa.

[87] Alexeeva N.B., Sokolova I.V. (2012). Iridaceae Juss. Catalogue of the Type Specimens of the Vascular Plants from Siberia and the Russian Far East Kept in the Herbarium of the Komarov Botanical Institute (LE).

[88] Boltenkov E.V. (2019). Typification and taxonomic notes on three irises names (Iridaceae) described from Central Asia. Phytotaxa.

[89] Doronkin V.M., Krasnoborov I.M., Artemov I.A. (2012). Iridaceae. Opredelitel Rasteniy Respubliki Altay [Key to Plants of the Altay Republic].

[90] Gubanov I.A. (1996). Conspectus of Flora of Outer Mongolia (Vascular Plants).

[91] Volis S., Depalle F., Khassanov F., Yusupov Z., Deng T. (2024). *Oncocyclus* irises: Phylogeny, evolutionary history and revised taxonomy based on complete chloroplast genome sequences. Plant Divers. Cent. Asia.

[92] Bunge A. (1852). Beitrag zur Kenntniss der Flora Russlands und der Steppen Central-Asiens.

[93] Boltenkov E.V. (2017). Taxonomic notes on hexapogon irises (Iridaceae). Phytotaxa.

[94] Baker J.G. (1876). A synopsis of the known species of *Iris*; I. Gard. Chron. New Ser..

[95] Rodionenko G.I. (1961). Rod Iris—Iris L. [The Genus Iris L.].

[96] Rodionenko G.I. (2009). A new system of the genus *Iris* (Iridaceae). Bot. Zhurn..

[97] Britton N.L., Brown A. (1913). An Illustrated Flora of the Northern United States, Canada and the British Possessions.

[98] Zakharyeva O.I. (1985). Chromosome numbers of some flowering plants from the Caucasus and Middle Asia. Bot. Zhurn..

[99] Bochantseva Z.P. (1969). Karyosystematics and morphology of the *Iris* species from the section *Regelia*. Introduktsija i Akklimatizatsija Rastenii.

[100] Wright C.H. (1904). Iris bismarckiana. Curtis’s Bot. Mag. Ser. 3.

[101] Sapir Y., Shmida A. (2002). Species concepts and ecogeographical divergence of *Oncocyclus* irises. Israel J. Plant Sci..

[102] Volis S., Zhang Y.-H., Deng T., Yusupov Z. (2023). Israeli *Oncocyclus* irises: Phylogenetic relationships and evolutionary history. Isr. J. Ecol. Evol..

[103] Avishai M., Zohary D. (1977). Chromosomes in the oncocyclus irises. Bot. Gaz..

[104] Boltenkov E.V. (2023). Taxonomic reinstatement of the endemic Chinese species *Iris thoroldii* (Iridaceae) from *I. potaninii* and reassessment of *I. zhaoana*. Plants.

