# Supplementary material for: Updated Taxonomy of Iris scariosa (Iridaceae) Inferred from Morphological and Chloroplast DNA Sequence Data with Remarks on Classification of Iris subg. Iris
Source: Plants (Basel). 2024 Aug 23;13(17):2349. doi: 10.3390/plants13172349 (PMC11397725; doi:10.3390/plants13172349)
Supplement: Supplementary file 1 [file plants-13-02349-s001.zip › Table S1_.doc]

**Table S1.** GenBank accession numbers for the *Iris* species sequences used in the study (see https://www.ncbi.nlm.nih.gov/, accessed on 15 March 2024).

| **Species** | **Accession numbers** |
| --- | --- |
| *I. afghanica* Wendelbo | NC081929 |
| *I. dihotoma* Pall. | OK448492 |
| *I. domestica* (L.) Goldblatt et Mabb. | MW039136 |
| *I. ensata* Thunb. | NC056173 |
| *I. gatesii* Foster | NC024936 |
| *I.* *germanica* L. | NC062594 |
| *I. goniocarpa* Baker | KY319646 |
| *I. haynei* Baker | MW476738 |
| *I. hoogiana* Dykes | NC085777 (= [OR774962](https://www.ncbi.nlm.nih.gov/nucleotide/OR774962.1?report=genbank&log$=nuclalign&blast_rank=1&RID=8W0RMZ13016)) |
| *I. imbricata* Lindl. | OR789640 |
| *I. laevigata* Fisch. | NC056176 |
| *I. lutescens* Lam. | OR752430 |
| *I. lycotis* Woronow | MW476739 |
| *I. pseudacorus* L. | NC056179 |
| *I. sichuanensis* Y.T.Zhao | [NC085782](https://www.ncbi.nlm.nih.gov/nucleotide/NC_085782.1?report=genbank&log$=nuclalign&blast_rank=22&RID=ZTTA9NVE016) (= OR789643) |
| *I. sprengeri* Siehe | OR789639 |
| *I. thoroldii* Baker  (sub *I. potaninii* var. *ionantha* Y.T.Zhao) | KY319645  HM574466 |
| *I. uniflora* Pall. ex Link | NC056183 |
